# Single-Cell Imaging Shows That the Transcriptional State of the HIV-1 Provirus and Its Reactivation Potential Depend on the Integration Site

**DOI:** 10.1128/mbio.00007-22

**Published:** 2022-06-16

**Authors:** Julie Janssens, Flore De Wit, Nagma Parveen, Zeger Debyser

**Affiliations:** a Molecular Virology and Gene Therapy, KU Leuven, Leuven, Belgium; b Department of Chemistry, Indian Institute of Technology, Kanpur, India; Johns Hopkins University School of Medicine; Albert Einstein College of Medicine

**Keywords:** HIV-1, LEDGF/p75, LEDGINs, integration site selection, single-cell HIV-1 imaging

## Abstract

Current antiretroviral treatment fails to cure HIV-1 infection since latent provirus resides in long-lived cellular reservoirs, rebounding whenever therapy is discontinued. The molecular mechanisms underlying HIV-1 latency are complex where the possible link between integration and transcription is poorly understood. HIV-1 integration is targeted toward active chromatin by the direct interaction with a host protein, lens epithelium-derived growth factor (LEDGF/p75). LEDGINs are small-molecule inhibitors of the LEDGF/p75-integrase (IN) interaction that effectively inhibit and retarget HIV-1 integration out of preferred integration sites, resulting in residual provirus that is more latent. Here, we describe a single-cell branched DNA imaging method for simultaneous detection of viral DNA and RNA. We investigated how treatment with LEDGINs affects the location, transcription, and reactivation of HIV-1 in both cell lines and primary cells. This approach demonstrated that LEDGIN-mediated retargeting hampered the baseline transcriptional state and the transcriptional reactivation of the provirus, evidenced by the reduction in viral RNA expression per residual copy. Moreover, treatment of primary cells with LEDGINs induced an enrichment of provirus in deep latency. These results corroborate the impact of integration site selection for the HIV-1 transcriptional state and support block-and-lock functional cure strategies in which the latent reservoir is permanently silenced after retargeting.

## INTRODUCTION

Combination antiretroviral therapy (cART) effectively controls HIV-1 by reducing active viral replication but does not cure the infection. A small fraction of integrated, intact HIV-1 proviruses remain present for decades in a transcriptionally silent state in long-lived memory cells. When cART is interrupted, stimulation of latently infected memory cells induces a viral rebound, making lifelong treatment a prerequisite for sustained viral suppression. The persistence of transcriptionally silent but replication-competent HIV-1, also referred to as the latent reservoir, represents one of the major challenges of HIV-1 cure research today.

Although the persistence of HIV-1 is intrinsically linked to integration of the provirus in the host genome, the impact of integration site selection on HIV-1 transcription, especially in the establishment of HIV-1 latency and reactivation, remains poorly understood (1). A link between HIV-1 integration and proviral expression was first postulated in 2001 by the Verdin lab after the observation of highly variable expression levels between transduced Jurkat clones (2). Afterward, the Bushman lab reported that low-level expression correlated with HIV-1 integration in centromeric heterochromatin regions and gene deserts (3). Nonetheless, the majority of integrated HIV-1 proviruses, including the transcriptionally silent ones, are found in actively transcribed and gene-dense regions (2, 4–6). This preferred integration pattern is dictated in large part by host factors, among which lens epithelium-derived growth factor (LEDGF/p75) (7–10) and cleavage and polyadenylation specificity factor 6 (CPSF6) (11–14) are important determinants of HIV-1 integration site selection.

LEDGF/p75 functions as a chromatin reader, tethering various proteins to active chromatin through recognition of methylated lysine 36 residues in histone 3 (H3K36me2 and H3K36me3) by its N-terminal PWWP domain (15). Furthermore, LEDGF/p75 encompasses two AT hook motifs for chromatin interaction (16, 17) and a C-terminal domain comprising the integrase binding domain (IBD) to which both HIV-1 integrase and cellular partners bind (18, 19). Depletion of LEDGF/p75 reduces integration (20) yet additionally shifts residual integration out of transcription units (7, 8, 21, 22). Small-molecule inhibitors that perturb the interaction between LEDGF/p75 and the HIV-1 integrase (IN) have been developed (23–25). These compounds are referred to as LEDGINs since they all bind the LEDGF/p75 pocket in HIV integrase (23). Since their initial discovery, compounds of different chemical nature but belonging to the same functional class have been described (26), including noncatalytic site integrase inhibitors (NCINIs) (27), allosteric integrase inhibitors (ALLINIs) (28), multimerization selective integrase inhibitors (MINIs) (29), and integrase-LEDGF allosteric inhibitors (INLAIs) (30).

LEDGINs potently inhibit HIV-1 replication by interfering with both the integration and virus assembly steps (24, 27, 31). LEDGINs can also affect HIV-1 latency and reactivation (21, 32, 33). After LEDGIN-mediated retargeting, the residual HIV-1 reservoir was shown to be more latent and less prone to reactivation (21). This result was corroborated recently using the LEDGIN GS-9822 (34), displaying activity in the low-nanomolar range, which increased immediate latency and reduced provirus reactivation at nanomolar concentrations in *in vitro*-infected T cell lines. Immediate latency refers to early silencing of HIV-1 expression, usually within 24 h prior to initiation of Tat-dependent HIV-1 transcription (35, 36). To understand how LEDGINs induce transcriptional silencing and prevent reactivation from latency, Vansant et al. studied the epigenetic landscape and chromatin environment surrounding the HIV-1 promoter of LEDGIN-retargeted provirus (33). Each single provirus was labeled with a unique barcode (20), thereby facilitating investigations into the impact of integration site selection on HIV-1 transcription. The authors confirmed the LEDGIN-mediated shift in integration away from H3K36me3, the epigenetic mark recognized by LEDGF/p75. An inverse correlation between the proximity to H3K36me3 and the transcriptional activity was reported, whereas residual expression was associated with proximity to (super)enhancers (33). The link between endogenous enhancers and HIV-1 expression is supported by a study from the Filion lab (37) wherein the authors have shown that the proximity of proviral sequences to enhancers also affects their response to latency reversal (37).

More recently, the significance of the HIV-1 integration profile on HIV-1 persistence *in vivo* has been supported by the study of the Yu lab characterizing the proviral reservoir in elite controllers (ECs) (38). ECs spontaneously control HIV-1 infection, as evidenced by undetectable levels of viremia, stable CD4-positive (CD4^+^) T cell counts, and no signs of clinical progression (39). The authors found that the latent reservoir in ECs was largely composed of intact proviral sequences that were integrated at distinct sites in the human genome, preferentially in noncoding and transcriptionally repressed regions, such as centromeres and genes that encode zinc finger proteins. No evidence was found for a preferential targeting of those silent regions during acute infection. More likely, proviral sequences integrated in actively expressed regions are gradually cleared by the immune system, while a deep latent reservoir is selected over time (40, 41), supporting the concept of deep latency as functional cure for HIV-1 (42).

Most studies so far that have investigated the link between integration site selection, transcription, and reactivation depend on ensemble analysis. Understanding of the link between HIV-1 integration site selection and transcription has been hampered by the lack of a method for simultaneous detection of integrated provirus and viral RNA expression. Puray-Chavez et al. optimized branched DNA (bDNA) imaging for simultaneous microscopic visualization of viral DNA and RNA in single cells. Here, we describe the use of this technology to study the impact of the HIV-1 integration site on the proviral transcription and reactivation potential using LEDGINs as a tool to retarget HIV-1 integration site selection in cells. Single-cell resolution of combined viral DNA (vDNA) and RNA (vRNA) detection showed how retargeting integration with LEDGINs shifted the three-dimensional location toward the inner nucleus, reduced transcription, and hampered reactivation of the provirus, demonstrating the direct link between proviral integration and transcription. The progressive enrichment of integrated provirus characterized by deep proviral latency over time in primary cell cultures may be similar to what happens in ECs, and it implies the selection of deeply latent provirus in primary cell cultures. Moreover, the silent phenotype observed after LEDGIN-mediated retargeting in SupT1 cell lines and primary cell culture models supports how retargeting integration can be used in a block-and-lock functional cure strategy in which the latent reservoir is permanently silenced.

## RESULTS

### A single-cell-based method to study HIV-1 latency and reactivation.

**FIG 1:**
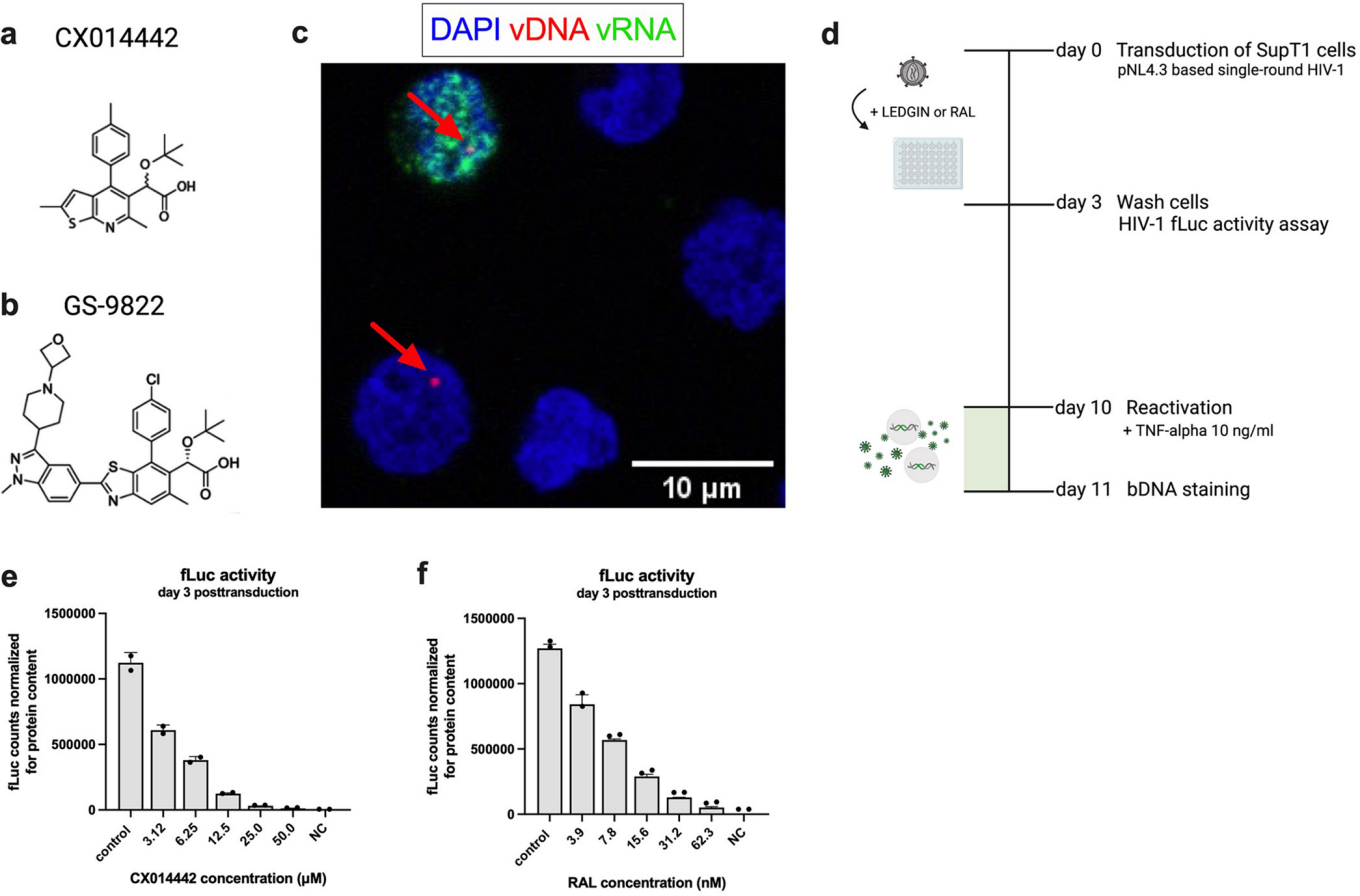
Branched DNA imaging to study HIV-1 latency and reactivation. (a, b) Compound structures of CX014442 (a) and GS-9822 (b). (c) After cell fixation and permeabilization, hybridization with target-specific Z probes allows fluorescence imaging of vDNA (red, as pointed by red arrow) and vRNA (green) with confocal microscopy, allowing us to differentiate between uninfected (vDNA^−^ vRNA^−^), actively infected (vDNA^+^ vRNA^+^), and latently infected cells (vDNA^+^ vRNA^−^). Nuclei are visualized with DAPI (blue). Scale bar represents 10 μm. (d) Timeline of the transduction and reactivation experiments. SupT1 cells were transduced with HIV-1 fLuc in the presence of LEDGIN (CX014442; GS-9822) or RAL. Three days posttransduction, the virus and compounds were washed away, and fLuc reporter expression was measured. Ten days posttransduction, the cells were reactivated with 10 ng/mL TNF-α. Twenty-four hours after reactivation, cells were fixed prior to bDNA staining and imaging. (e, f) SupT1 cells were transduced with single-round HIV-1 fLuc in the presence of CX014442 (e) or RAL (f). fLuc activity was measured 3 days posttransduction and normalized for the total protein content. The normalized fLuc activity is shown as mean ± standard deviation from one representative experiment (*n* = 3), NC, nontransduced negative control.

LEDGINs are small molecules (Fig. 1a and b) that interfere with the interaction between HIV-1 IN and LEDGF/p75 and retarget integration (21, 24, 33). Previously, we reported that LEDGIN-mediated retargeting of HIV-1 integration increases immediate HIV-1 latency and reduces reactivation (21, 33). We now optimized a bDNA fluorescent *in situ* hybridization (FISH) imaging method, previously described by Puray-Chavez et al. (43), to study the effect of integration site retargeting on HIV-1 latency and reactivation at the single-cell level. Simultaneous detection of vDNA and vRNA allows discrimination between actively HIV-1-infected cells (vDNA^+^, vRNA^+^); latently infected cells (vDNA^+^, vRNA^−^), and uninfected cells (vDNA^−^, vRNA^−^), as illustrated in Fig. 1c.

**FIG 2:**
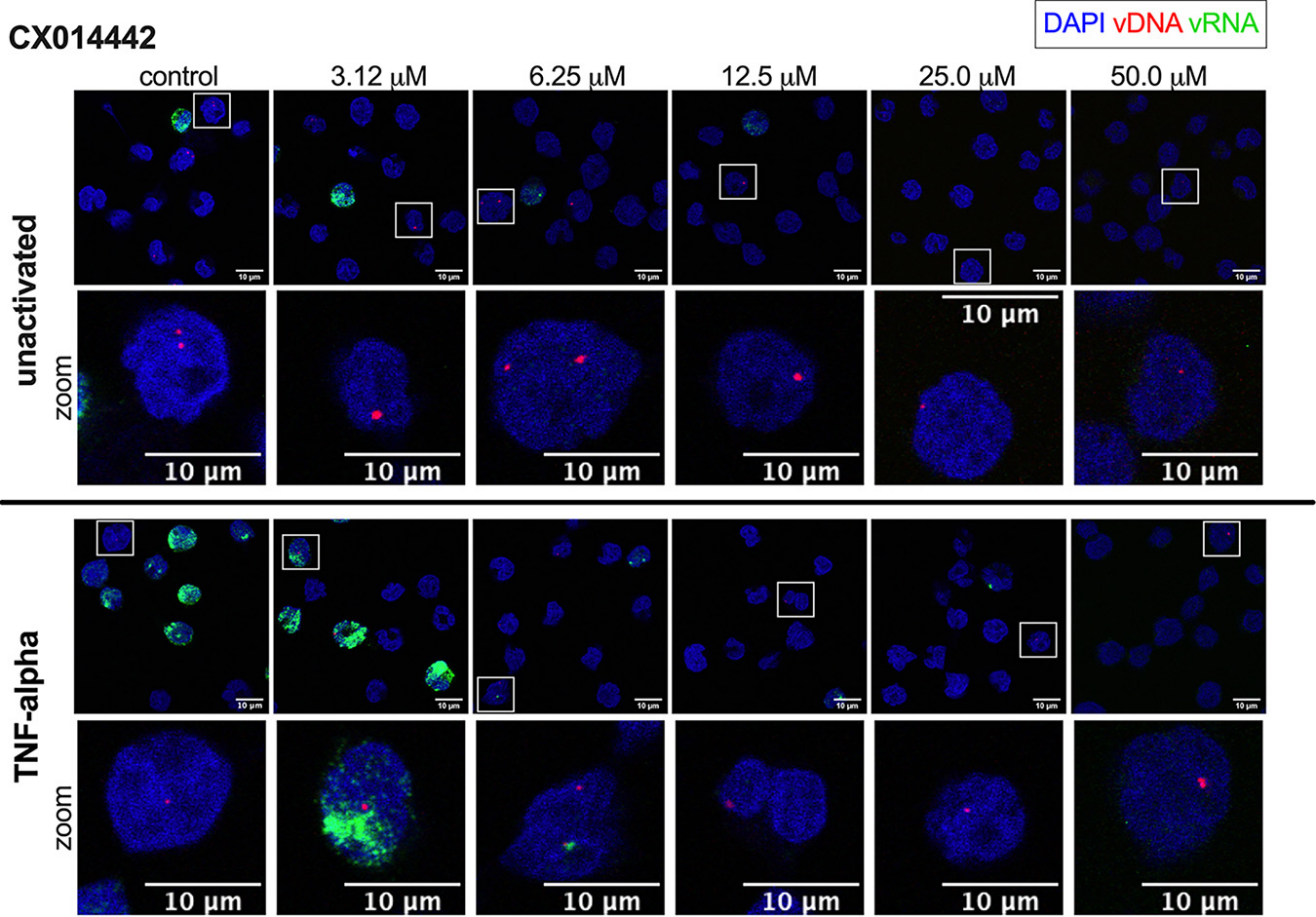
bDNA imaging of LEDGIN CX014442-treated cells. SupT1 cells were transduced with single-round HIV-1 and reactivated 10 days posttransduction with 10 ng/mL TNF-α. Twenty-four hours after reactivation, the cells were fixed to visualize vDNA (red) and vRNA (green) with bDNA imaging in cells treated with 0 to 50 μM LEDGIN CX014442. Unactivated cells (top) and TNF-α-reactivated cells (bottom) are shown. For better visualization of the vDNA spots, a zoomed image of the cell highlighted by the square box is shown for each condition. Nuclei are stained with DAPI (blue), and scale bars represent 10 μm.

**FIG 3:**
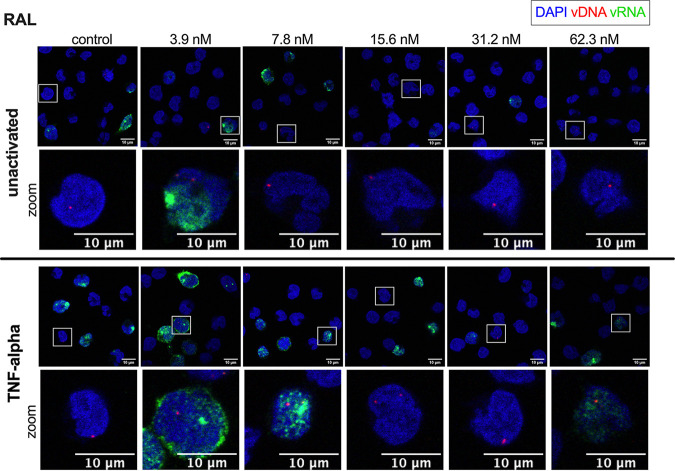
bDNA imaging of RAL-treated cells. SupT1 cells were transduced with single-round HIV-1 and reactivated 10 days posttransduction with 10 ng/mL TNF-α. Twenty-four hours after reactivation, the cells were fixed to visualize vDNA (red) and vRNA (green) with bDNA imaging in cells treated with 0 to 62.3 nM RAL. Unactivated cells (top) and TNF-α-reactivated cells (bottom) are shown. For better visualization of the vDNA spots, a zoomed image of the cell highlighted by the square box is shown for each condition. Nuclei are stained with DAPI (blue), and scale bars represent 10 μm.

SupT1 cells were transduced with 8.5 × 10^4^ pg p24 replication-deficient HIV-1-fLuc and cultured for 3 days in the presence of different concentrations of LEDGIN CX014442. The 50% inhibitory concentration (IC_50_) of CX014442 against the single-round HIV-1 fLuc was 9.6 μM (average IC_50_ values from 3 independent experiments, 11.8 ± 3.3 μM, 8.5 ± 2.1 μM, and 8.7 ± 2.9 μM) (see Fig. S1a in the supplemental material). LEDGIN treatment was compared to RAL, for which the IC_50_ was 12.9 nM (average IC_50_ from 3 independent experiments, 12.0 ± 1.7 nM, 13.0 ± 1.7 nM, and 13.6 ± 1.6 nM) (Fig. S1b). The timeline of the transduction and reactivation experiments is outlined in Fig. 1d. Three days posttransduction, the inhibitory effect of CX014442 and RAL on HIV-1 infectivity was confirmed by measuring the fLuc reporter expression (Fig. 1e and f, fLuc activity). The cells were washed twice to remove cell-free virus and compound and further cultured in the absence of compound to allow reversion to a latent state. After 10 days, latently infected cells were either left untreated or reactivated with 10 ng/mL tumor necrosis factor alpha (TNF-alpha). After 24 h reactivation, the cells were fixed to perform bDNA imaging, shown in Fig. 2 and 3.

10.1128/mbio.00007-22.1 FIG S1 IC_50_ values of antivirals in bDNA imaging experiments. (a to d) The average IC_50_, calculated by nonlinear regression of a dose-response curve, was calculated from 3 (CX014442 and RAL) (a and b), 4 (LEDGIN GS-9822) (c), or 2 (CX014442 [2]) (d) independent experiments. The mean fLuc activity ± standard deviation is shown normalized for the total protein content. Download FIG S1, TIF file, 0.4 MB. Copyright © 2022 Janssens et al. 2022 Janssens et al. https://creativecommons.org/licenses/by/4.0/ This content is distributed under the terms of the Creative Commons Attribution 4.0 International license.

### CX014442 and RAL both reduce HIV-1 integration at the single-cell level.

**FIG 4:**
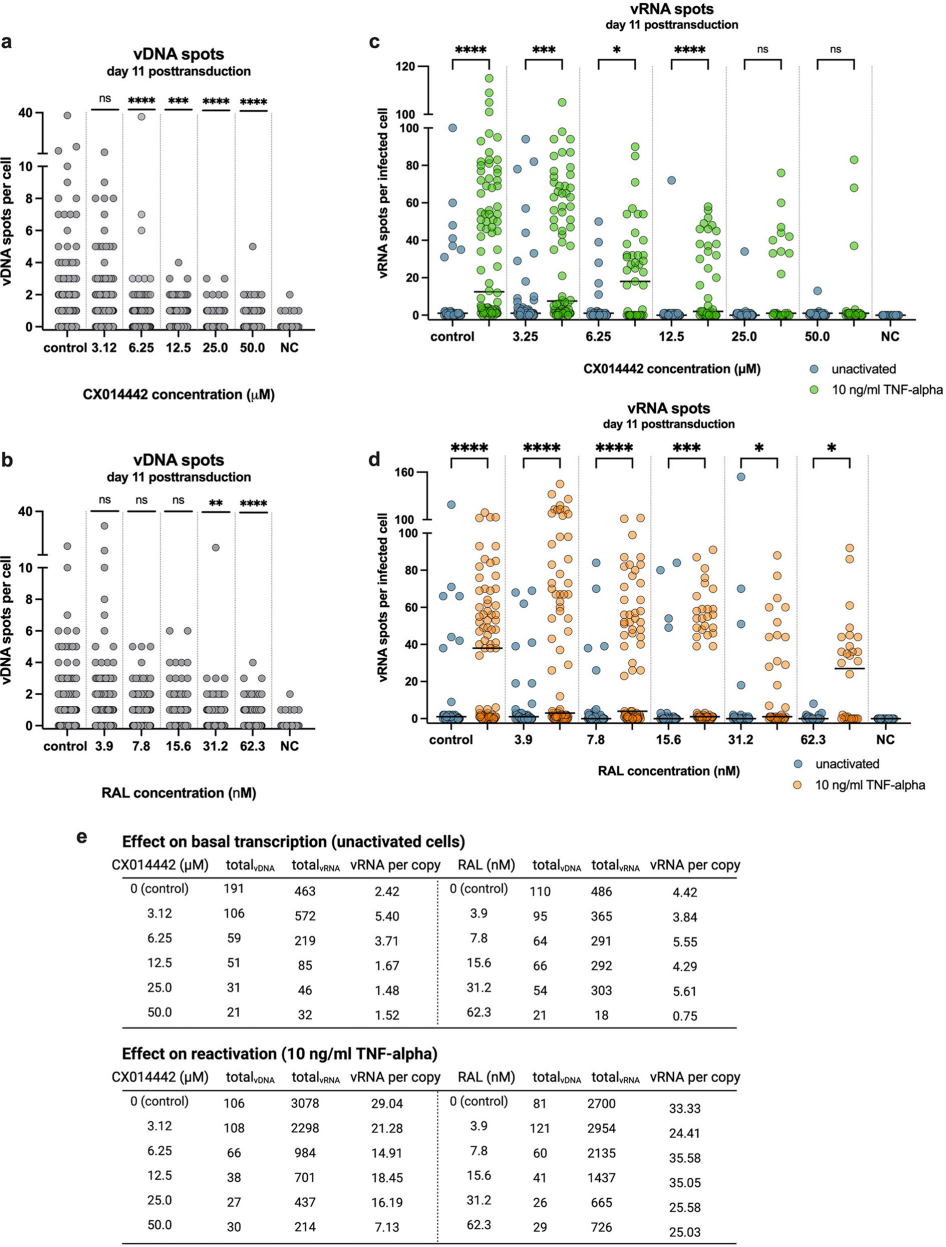
LEDGIN CX014442, but not RAL, reduces HIV-1 transcription and reactivation. (a, b) The number of vDNA spots per cell, measured with bDNA imaging 11 days posttransduction after treatment with CX014442 (a) or RAL (b) in SupT1 cells. Pooled data from the unactivated and reactivated cells are shown. The numbers of cells imaged and analyzed per condition are shown in Table S2b and c in the supplemental material. A Kruskal-Wallis test was used to test for statistical significance compared to the control condition: **, *P* < 0.01; ***, *P* < 0.001; ****, *P* < 0.0001. NC, nontransduced negative control. (c, d) The numbers of vRNA spots per infected cell are shown for the unactivated and reactivated cells, measured with bDNA imaging 11 days posttransduction in the presence of CX014442 (c) and RAL (d). The numbers of cells imaged and analyzed per condition are shown in Table S2b and c. The median number of vRNA spots per cell is indicated with a horizontal line. A Kruskal-Wallis test was used to test for statistical significance between the unactivated and reactivated cells of each condition: *, *P* < 0.05; ***, *P* < 0.001; ****, *P* < 0.0001; and ns, nonsignificant. NC, nontransduced negative control (e) Viral RNA expression per residual copy was calculated in the presence of increasing concentrations of CX014442 or RAL by dividing the number of vRNA spots by the number of vDNA spots for the unreactivated cells (top) and reactivated cells (bottom).

After bDNA imaging in SupT1 cells (Fig. 2 and 3), quantification of the vDNA spots per single cell was performed to evaluate the inhibitory effect of CX014442 and RAL on HIV-1 integration. The vDNA probes cannot discriminate between integrated and unintegrated vDNA. However, passaging transduced cells eliminates unintegrated vDNA over time to undetectable levels (44), while integrated vDNA will remain stably associated with the cell (Fig. S2). As such, all vDNA spots detected with bDNA imaging 11 days posttransduction are expected to be stably integrated. To directly compare CX014442 with RAL, equivalent concentrations were calculated based on the IC_50_ value of each inhibitor (ranging from 0.3 to 5.2× IC_50_). As expected, reactivation of the cells 10 days posttransduction with TNF-α did not significantly alter the level of vDNA spots (Fig. S3a and b). Therefore, the vDNA spots detected in the unactivated and TNF-α-treated cells were pooled for each concentration (Fig. 4a and b). For both inhibitors, a significant reduction in vDNA spots was observed with increasing concentrations of the inhibitor (Fig. 4a and b), corroborating that both CX014442 and RAL reduce HIV-1 integration (26, 45).

10.1128/mbio.00007-22.2 FIG S2 Detection of the number of integrated vDNA copies (a, b) Integrated copies were determined in the presence of CX014442 on day 11 posttransduction with Alu-LTR qPCR normalized to CCR5, presented as mean ± standard deviation (a) and bDNA imaging (b). The average number of vDNA spots per cell is shown as mean ± SEM. Download FIG S2, TIFF file, 0.2 MB. Copyright © 2022 Janssens et al. 2022 Janssens et al. https://creativecommons.org/licenses/by/4.0/ This content is distributed under the terms of the Creative Commons Attribution 4.0 International license.

10.1128/mbio.00007-22.3 FIG S3 TNF-alpha exposure does not affect vDNA detection. The number of vDNA spots per cell, measured with bDNA imaging 11 days posttransduction, is shown in the presence of CX014442 (a) and RAL (b). The numbers of vDNA spots are shown separately for either the unactivated or reactivated cells. The number of cells analyzed per condition is shown in Table S2a. A Kruskal-Wallis test was used to test for statistically significant differences. ns, nonsignificant. Download FIG S3, TIFF file, 0.5 MB. Copyright © 2022 Janssens et al. 2022 Janssens et al. https://creativecommons.org/licenses/by/4.0/ This content is distributed under the terms of the Creative Commons Attribution 4.0 International license.

10.1128/mbio.00007-22.8 TABLE S2 Number of cells imaged and analyzed per condition. The number of cells imaged and analyzed per condition is listed per imaging experiment for the vDNA and vRNA analysis. Download Table S2, JPG file, 0.5 MB. Copyright © 2022 Janssens et al. 2022 Janssens et al. https://creativecommons.org/licenses/by/4.0/ This content is distributed under the terms of the Creative Commons Attribution 4.0 International license.

### Treatment with LEDGIN CX014442 shifts the three-dimensional location of the integrated provirus.

It is well-known that LEDGIN treatment retargets integration out of the preferred integration sites, away from H3K36me3 (21, 33, 34). Most (5, 21, 46), but not all (14), studies claim that the location of retargeted proviruses either induced by depletion of LEDGF/p75 or by LEDGIN treatment is shifted toward the inner nuclear compartment. Here, we used bDNA imaging to investigate the three-dimensional distribution of the provirus after CX014442 and RAL treatment. To this end, SupT1 cells were segmented based on an intensity threshold of the DAPI (4′,6-diamidino-2-phenylindole) signal, and the outer boundary was defined as nuclear boundary. Quantitative analysis of the vDNA spots was performed with a MATLAB routine, and the minimum distance between the particle and the nuclear boundary was calculated using the Euclidean algorithm. As shown in Fig. S4a, the location of the integrated proviruses tends to be dose-dependently shifted farther away from the nuclear boundary in the CX014442-treated cells; however, it is not statistically different. On the contrary, the location of the integrated proviruses in the RAL-treated cells is similar to the location observed in the control condition except for the cells treated with 30 nM RAL (Fig. S4b). Only the cells with one integrated copy were included (see Fig. S4c for the number of cells analyzed per condition). We hypothesized that additional copies in cells with a multiplicity of infection (MOI) of >1 may deviate from the bona fide integration profile. Furthermore, this selection allows comparison of similar numbers of integrated proviruses (Fig. S4c).

10.1128/mbio.00007-22.4 FIG S4 Treatment with LEDGIN CX014442 shifts the location of the integrated provirus. (a, b) Distance (μm) of the integrated proviruses (vDNA spots) from the nuclear boundary in the presence of CX014442 (a) and RAL (b). Means with 95% confidence intervals are shown overlapping individual points from one representative experiment (*n* = 2). Only the cells with one integrated provirus were included in the analysis. (c) Number of cells with one integrated provirus analyzed per condition. Download FIG S4, TIF file, 0.3 MB. Copyright © 2022 Janssens et al. 2022 Janssens et al. https://creativecommons.org/licenses/by/4.0/ This content is distributed under the terms of the Creative Commons Attribution 4.0 International license.

### CX014442, but not RAL, reduces HIV-1 transcription and reactivation.

LEDGIN CX014442 has previously been shown to reduce transcription and reactivation of the residual provirus (21, 33). To investigate the impact of retargeting integration, the vRNA expression level was analyzed at the single-cell level with bDNA imaging and compared to cells treated with RAL (Fig. 2 and 3). All uninfected cells, the proportion of which increased with higher inhibitor concentrations, were left out of the analysis to account for the fact that both CX014442 and RAL reduce HIV-1 integration. Only cells with vDNA and/or vRNA copies were included. Quantitative analysis revealed that the number of vRNA spots per infected cell decreased in a dose-dependent manner after CX014442 treatment in the unactivated cells (Fig. 4c, blue spots) indicating that CX014442 reduced basal HIV-1 transcription. In cells that were not treated with CX014442, reactivation with TNF-α resulted in a significant, on average, 13-fold increase in vRNA spots per infected cell compared to the unactivated cells (*P* < 0.0001) (Fig. 4c, green spots). After CX014442 treatment, a dose-dependent reduction in HIV-1 reactivation was evidenced, indicated by a relative decrease in vRNA spots per infected cell. In the cells treated with 25.0 and 50.0 μM CX014442, except for the presence of a few residual high vRNA expressors, reactivation did not lead to a significant increase in vRNA spots per infected cell, indicating that retargeting significantly hampers HIV-1 reactivation. Basal HIV-1 transcription was also reduced after RAL treatment (Fig. 4d, blue spots), albeit to a lesser extent than the reduction observed after CX014442 treatment. In contrast to CX014442 treatment, treatment with high concentrations of RAL still allowed a significant reactivation of HIV-1 expression (*P* < 0.05 for 32.2 and 62.3 nM RAL) (Fig. 4d).

To corroborate that the dose-dependent reduction in HIV-1 transcription and reactivation after CX014442 treatment was not solely the result of reduced HIV-1 infectivity, we calculated the level of vRNA expression per residual copy (total number of vRNA spots/total number of vDNA spots). In cells treated with 6.25 μM CX014442, the numbers of both vDNA and vRNA decreased; still, we observed a net increase in vRNA expression per copy from 2.42 (control) to 3.71 (6.25 μM), indicating that the reduction in vDNA spots was more pronounced than reduction in vRNA spots (Fig. 4e, top left). For cells treated with 12.5, 25.0, and 50.0 μM CX014442, this ratio decreased from 2.42 (control) to 1.67, 1.48, and 1.52, respectively. No decrease in vRNA expression per copy was observed in the cells treated with RAL except at the highest concentration (Fig. 4e, top right). In addition, a dose-dependent decrease in vRNA expression per copy was observed after reactivation in the cells treated with CX014442, but not in the RAL-treated cells (Fig. 4e, bottom). Altogether, these data indicate that CX014442 not only inhibits HIV-1 integration but additionally impairs HIV-1 transcription and reactivation from latency. Since RAL fails to reduce HIV-1 transcription and reactivation, this effect is not merely the result of reduced infectivity.

To evaluate the effect of CX014442 on HIV-1 transcription and reactivation in more detail, the data were fit using a negative binomial probability distribution. Similar to the Poisson distribution, the negative binomial distribution is used to model count integer variables (here, the number of vRNA spots per cell). The negative binomial distribution is, however, more appropriate when there is a high degree of overdispersion in outcome variable (the number of vRNA spots, i.e., when mean and variance differ significantly from each other). According to the model outlined in Table S1, the number of vRNA spots (dependent variable) was predicted by the concentration of CX014442 used and by reactivation of cells (independent variables). In the unactivated cells treated with 6.25 μM CX014442, the odds ratio (OR) was 0.51, which equals a 49% decrease in vRNA spots compared to the control cells not treated with CX014442. In parallel, treatment with 12.5, 25.0, and 50.0 μM CX014442 further decreased the OR by 73, 81, and 86%, respectively. For cells treated with 7.8, 15.6, and 31.2 nM, we observed a more modest decrease of 36, 34, and 15% in OR, respectively. In the cells treated with 62.3 nM RAL, we obtained a decrease of 89%, which is similar to the decrease observed after treatment with 50.0 μM CX014442 (86%). For cells not treated with CX014442, reactivation with TNF-α increased the OR, on average, 400% compared to the unactivated cells. To determine the OR of the reactivated cells treated with CX014442, the OR was multiplied by the interaction term between the concentration and reactivation. For example, the OR for reactivated cells that were treated with 6.25 μM CX014442 was determined at 0.66 (0.51 × 1.30), which equals a 34% decrease in OR compared to the reactivated cells not treated with CX014442. Compared to the untreated cells that were reactivated, the OR further decreased by 51, 58, and 83% after 12.5, 25.0, and 50.0 μM CX014442 treatment, respectively. Treatment with 7.8, 15.6, 31.2 and, 62.3 nM RAL decreased the OR for reactivated cells by 13, 29, 50, and 30%, respectively. Only for the cells treated with 62.3 nM RAL, the interaction between concentration and reactivation was significant, suggesting that the level of inhibition was different for unactivated cells (89% decrease in OR) and reactivated cells (30% decrease in OR). In contrast to CX014442, RAL only modestly inhibited reactivation, but no dose-dependent inhibition was observed, implying that this is likely a result of reduced HIV-1 infectivity upon RAL treatment. In conclusion, based on the negative binomial regression model, only CX014442 severely impaired HIV-1 transcription and reactivation in a dose-dependent manner.

10.1128/mbio.00007-22.7 TABLE S1 LEDGIN CX014442 dose-dependently reduces vRNA expression and inhibits reactivation. Results of negative binomial regression analysis of the vRNA spots per infected cell. The parameter estimates are reported, along with the standard error, *P* value, and OR of each parameter. The ORs of vRNA spots per infected cell are shown for unactivated cells (top), the ORs of the interaction term between concentration and reactivation are shown in the middle panel, and the recalculated ORs are shown for the reactivated cells (bottom). OR, odds ratio. Download Table S1, JPG file, 0.4 MB. Copyright © 2022 Janssens et al. 2022 Janssens et al. https://creativecommons.org/licenses/by/4.0/ This content is distributed under the terms of the Creative Commons Attribution 4.0 International license.

### LEDGIN GS-9822 reduces HIV-1 transcription and reactivation at nanomolar concentrations.

**FIG 5:**
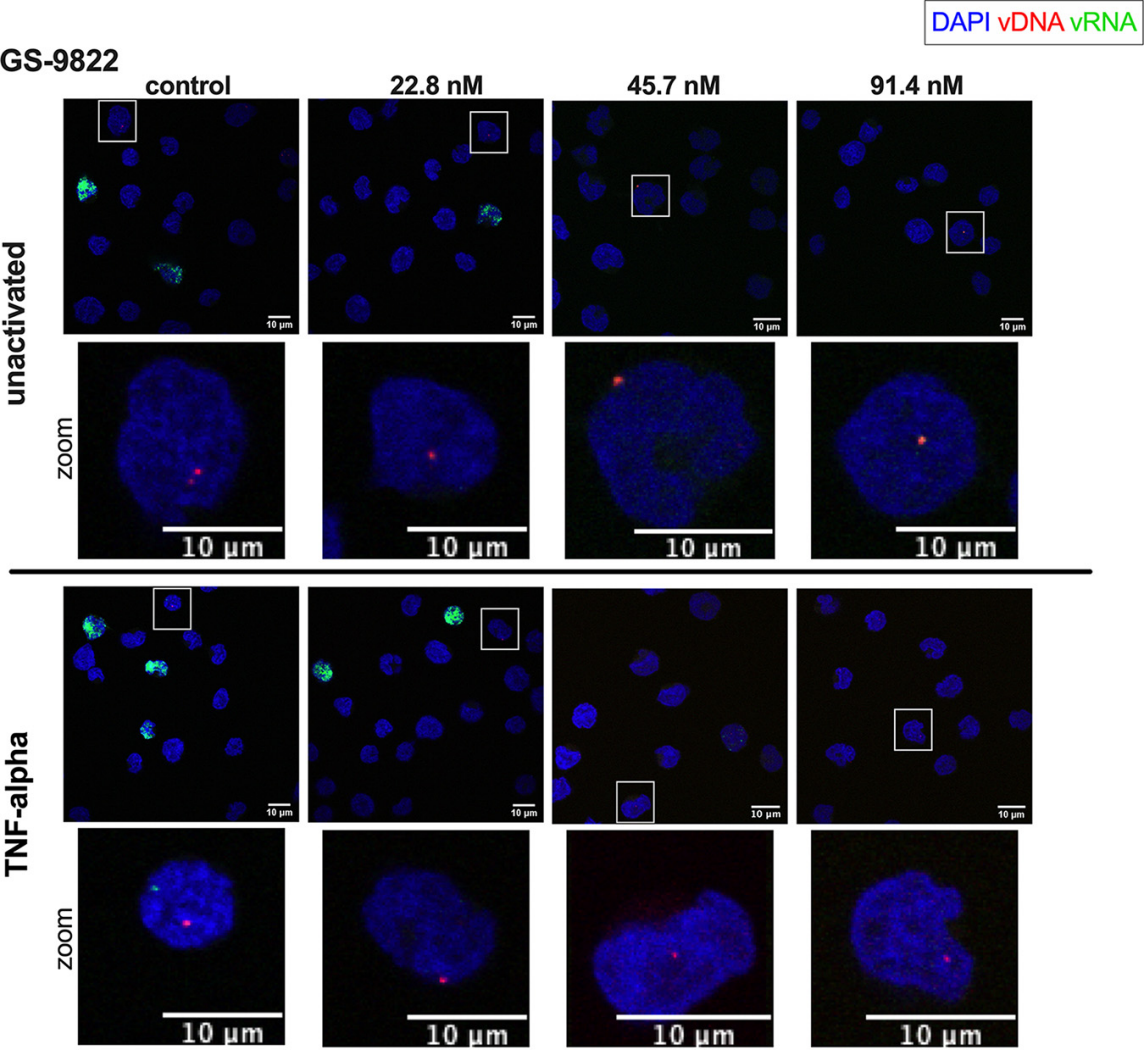
bDNA imaging of GS-9822-treated cells. SupT1 cells were transduced with single-round HIV-1 and reactivated 10 days posttransduction with 10 ng/mL TNF-α. Twenty-four hours after reactivation, the cells were fixed to visualize vDNA (red) and vRNA (green) with bDNA imaging in cells treated with increasing concentrations of GS-9822. Unactivated cells (top) and TNF-α-reactivated cells (bottom) are shown. For better visualization of the vDNA spots, a zoomed image of the cell highlighted by the square box is shown for each condition. Nuclei are stained with DAPI (blue), and scale bars represent 10 μm.

**FIG 6:**
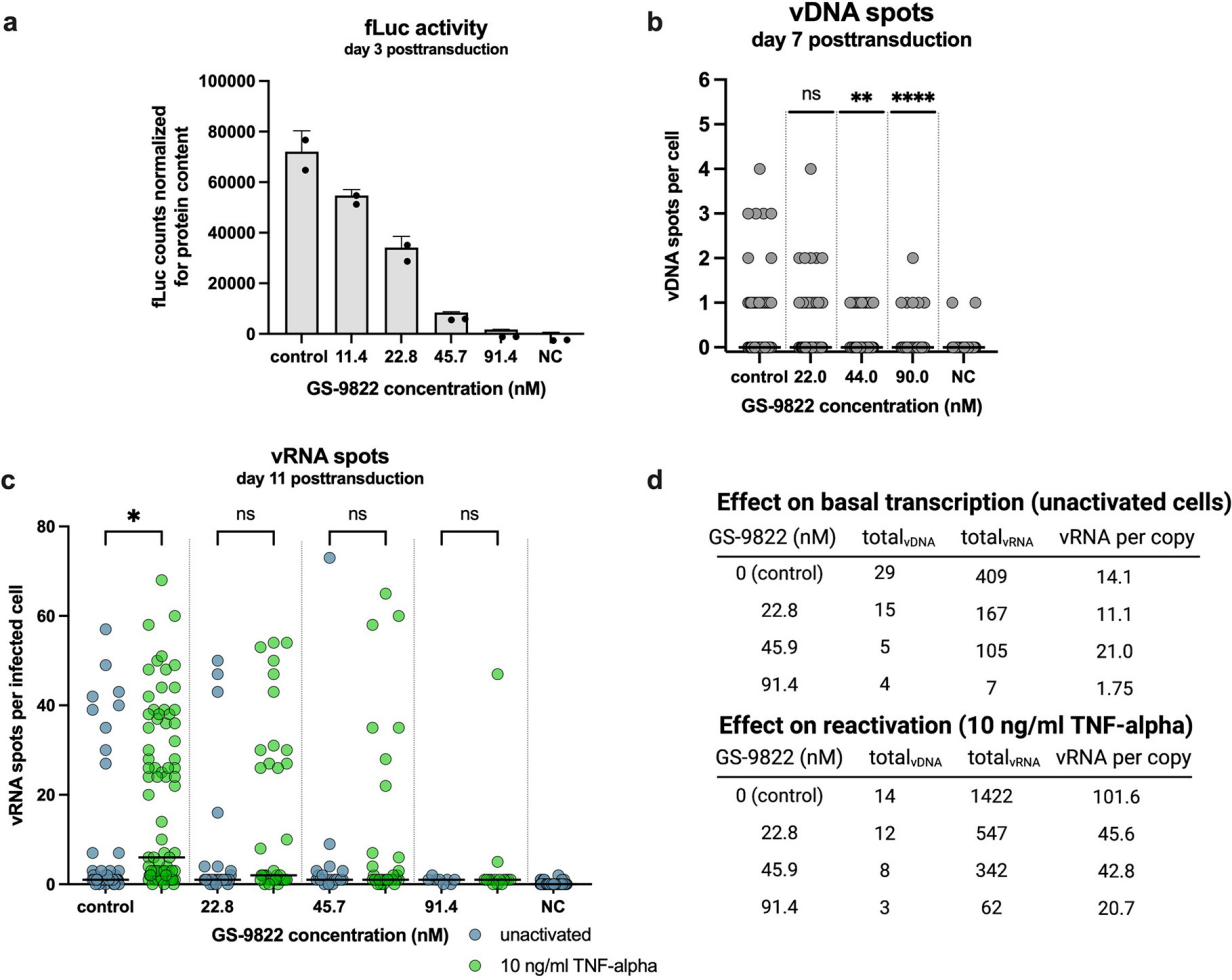
LEDGIN GS-9822 reduces HIV-1 transcription and reactivation at nanomolar concentrations. (a) SupT1 cells were transduced with single-round HIV-1 expressing firefly luciferase (fLuc) in the presence of GS-9822. The fLuc activity was measured 3 days posttransduction and normalized for the total protein content. The normalized fLuc activity is shown as mean ± standard deviation from one representative experiment (*n* = 2), performed in duplicate. NC, nontransduced negative control. (b) The numbers of vDNA spots per cell, measured with bDNA imaging 11 days posttransduction, are shown in the presence of GS-9822. Pooled data from the unactivated and reactivated cells are shown. The numbers of cells that were imaged and analyzed per condition are shown in Table S2d in the supplemental material. A Kruskal-Wallis test was used to test for statistically significant differences compared to the control condition: **, *P* < 0.01, and ****, *P* < 0.0001. NC, nontransduced negative control. (c) The numbers of vRNA spots per infected cell are shown for the unactivated and reactivated cells, measured with bDNA imaging 11 days posttransduction in the presence of LEDGIN GS-9822. The median number of vRNA spots per cell is indicated with a horizontal line. A Kruskal-Wallis test was used to test for statistically significant differences between the unactivated and reactivated cells of each condition: *, *P* < 0.05; ns, nonsignificant. NC, nontransduced negative control. The numbers of cells that were imaged and analyzed per condition are shown in Table S2d. (d) Viral RNA expression per residual copy was calculated in the presence of increasing concentrations of GS-9822 by dividing the number of vRNA spots by the number of vDNA spots for the unreactivated cells (top) and reactivated cells (bottom).

The effects observed with CX014442 were recently compared to those of the more potent LEDGIN GS-9822 for its inhibitory effects on HIV-1 replication and reactivation (34). GS-9822 displayed similar effects on HIV-1 retargeting, immediate latency, and latency reactivation, but at nanomolar concentrations in ensemble analyses (34). The observed effects being shared between two distinct LEDGINs of widely divergent potency underscores the class-specific effect of these molecules, which is not merely a result of cytotoxicity, for instance. Here, we investigated the addition of GS-9822 with bDNA imaging (Fig. 5). The IC_50_ of GS-9822, determined with the fLuc reporter readout, was 17.6 nM (average IC_50_ values from 4 independent experiments, 16.2 ± 3.4 nM, 17.8 ± 3.9 nM, 18.9 ± 4.8 nM, and 17.4 ± 4.4 nM) (Fig. S1c). Similar to CX014442, concentrations of GS-9822 equivalent to 1.3 to 5.2 × the IC_50_ were used. GS-9822 reduced HIV-1 infectivity (Fig. 6a) and HIV-1 integration (Fig. 6b). In addition, GS-9822 severely inhibited HIV-1 transcription and reactivation, even at nanomolar concentrations, as illustrated by the strong decrease in the number of vRNA spots per infected cell (Fig. 6c). In addition, a dose-dependent decrease in vRNA expression per copy was observed after reactivation in the cells treated with GS-9822 (Fig. 6d).

Multiple linear regression analysis calculated the OR of the vRNA spots per infected cell at 0.72 after treatment with 22.8 nM GS-9822 in the unactivated cells. This equals a 28% decrease in vRNA spots compared to the control cells not treated with GS-9822. In parallel, treatment of the unactivated cells with 45.7 and 91.4 nM GS-9822 was associated with a 43% and 91% reduction in OR. For the reactivated cells, the reduction in OR was 25, 38, and 77% in the cells treated with 22.8, 45.7, and 91.4 nM GS-9822, respectively (Table S3). In conclusion, the effects on HIV-1 transcription and reactivation observed with GS-9822 corresponded to the effects observed with CX014442; however, they were demonstrated at nanomolar concentrations.

10.1128/mbio.00007-22.9 TABLE S3 LEDGIN GS-9822 dose-dependently reduces vRNA expression at nanomolar concentrations. Results of negative binomial analysis of the vRNA spots per infected cell. The parameter estimates are reported, along with the standard error, *P* value, and OR of each parameter. The ORs of vRNA spots per infected cell are shown for unactivated cells (top), the ORs of the interaction term between concentration and reactivation are shown in the middle panel, and the recalculated ORs are shown for the reactivated cells (bottom). OR, odds ratio. Download Table S3, JPG file, 0.2 MB. Copyright © 2022 Janssens et al. 2022 Janssens et al. https://creativecommons.org/licenses/by/4.0/ This content is distributed under the terms of the Creative Commons Attribution 4.0 International license.

### LEDGIN treatment in primary cells hampers HIV-1 transcription and reactivation from latency.

**FIG 7:**
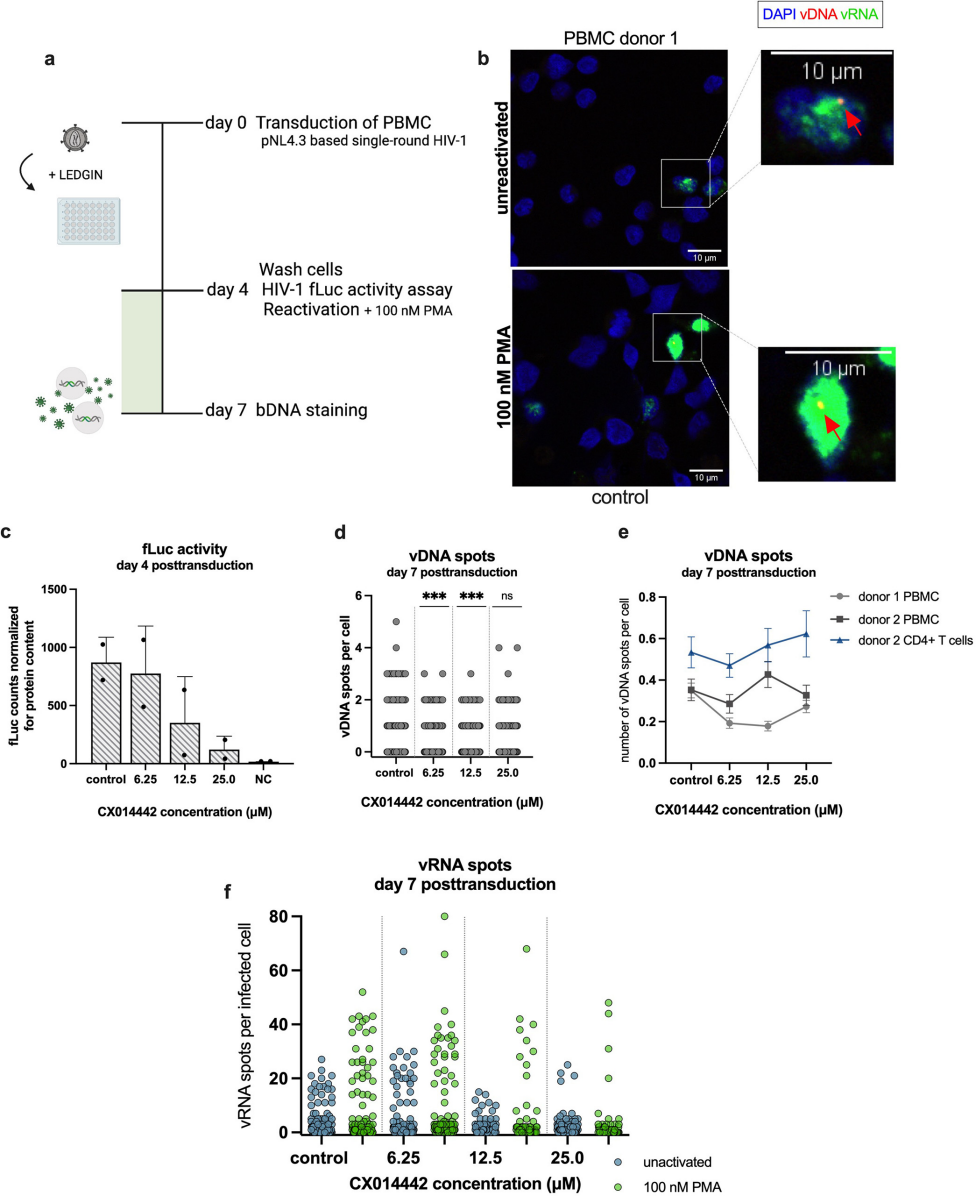
LEDGIN CX014442 treatment in primary cells hampers HIV-1 transcription and reactivation from latency. (a) Peripheral blood mononuclear cells (PBMCs, donor 1) were transduced with single-round HIV-1 expressing firefly luciferase (fLuc) in the presence of LEDGIN CX014442. On day 4 posttransduction, half of the cells were activated with 100 nM PMA, and samples were harvested for bDNA imaging 7 days posttransduction. (b) PBMCs were transduced with single-round HIV-1 and reactivated 4 days posttransduction with 100 nM PMA. On day 7, 72 h after reactivation, the cells were fixed to visualize vDNA (red, as pointed by red arrow) and vRNA (green) with bDNA imaging. Representative images are shown for the control condition. Nuclei are stained with DAPI (blue), and scale bars represent 10 μm. (c) fLuc activity was measured 4 days posttransduction and normalized for the total protein content. The normalized fLuc activity is shown as mean ± standard deviation from one representative experiment (*n* = 3), performed in duplicate. (d) The number of vDNA spots per cell, measured with bDNA imaging 7 days posttransduction, is shown in the presence of CX014442. Pooled data from the unactivated and reactivated cells are shown. The numbers of cells that were imaged and analyzed per condition are shown in Table S2e in the supplemental material. A Kruskal-Wallis test was used to test for statistically significant differences compared to the control condition: ***, *P* < 0.001. (e) The average number of vDNA spots per cell is shown as mean ± SEM. (f) The numbers of vRNA spots per infected cell are shown for the unactivated and reactivated cells, measured with bDNA imaging 7 days posttransduction in the presence of CX014442. The numbers of cells that were imaged and analyzed per condition are shown in Table S2e.

To evaluate the effect of LEDGINs on HIV-1 expression and reactivation in a more translational setting, bDNA imaging in primary peripheral blood mononuclear cells (PBMCs) and CD4^+^ T cells was performed. Primary PBMCs and CD4^+^ T cells were isolated from buffy coats from two healthy donors and transduced with 1.7 × 10^6^ pg replication-deficient HIV-1-fLuc. After culturing the cells for 4 days in the presence of increasing concentrations of CX014442 (6.25 to 25.0 μM), the compound and virus were washed away, and the cells were activated with 100 nM phorbol myristate acetate (PMA), outlined in Fig. 7a. On day 7 posttransduction, 72 h after activation, the cells were fixed to perform bDNA imaging (Fig. 7b). Reduced HIV-1 infectivity was confirmed upon addition of LEDGINs at day 4 posttransduction (Fig. 7c). LEDGINs significantly reduced the number of vDNA spots per cell when treated with 6.25 or 12.5 μM CX014442. Unexpectedly, no further decrease in vDNA spots was observed after treatment with 25.0 μM CX014442 (Fig. 7d). This observation was confirmed in primary cells from another donor, as illustrated by the increase in average vDNA spots per cell with increasing concentrations of CX014442 in both PBMCs and CD4^+^ T cells (Fig. 7e). Despite an increase in vDNA spots in the cells treated with 25.0 μM CX014442, HIV-1 expression was severely reduced in those cells, as indicated by a 7-fold-reduced fLuc activity (measured at day 4 posttransduction) (Fig. 7c) and by the reduction in vRNA expression in both unactivated and reactivated PBMCs (measured at day 7 posttransduction) (Fig. 7f). In the unactivated cells, treatment with CX014442 decreased the OR of vRNA spots with a maximum of 53% (Table S4a). For reactivated cells, treatment with 12.5 and 25.0 μM CX014442 reduced the number of vRNA spots by 26 and 61%, respectively, corroborating that CX014442 reduced HIV-1 expression and impaired latency reactivation in primary cells. Although vRNA expression was severely reduced in the cells treated with 25.0 μM CX014442, the enrichment of vDNA spots may point toward enrichment of deeply latent proviruses. Increased proviral latency and reduced reactivation after LEDGIN treatment was confirmed in PBMCs and CD4^+^ T cells isolated from a second donor (Fig. S5; Table S4b).

10.1128/mbio.00007-22.5 FIG S5 LEDGIN CX014442 reduces transcription and reactivation in primary cells. (a) PBMC and CD4^+^ T cells (donor 2) were transduced with single-round HIV-1 expressing firefly luciferase (fLuc) in the presence of CX014442. fLuc activity was measured 4 days posttransduction and normalized for the total protein content. The normalized fLuc activity from one representative experiment is presented as mean ± standard deviation. (b, c) The number of vDNA spots per cell, measured with bDNA imaging 7 days posttransduction, is shown in the presence of CX014442 for PBMC (b) and CD4^+^ T cells (c). Pooled data from the unactivated and reactivated cells are shown. The numbers of cells that were imaged and analyzed per condition are shown in Table S2f and g. A Kruskal-Wallis test was used to test for statistically significant differences compared to the control condition: ns, nonsignificant. (d, e) PBMCs and CD4^+^ T cells were transduced with single-round HIV-1 and reactivated 4 days posttransduction with 100 nM PMA (PBMCs) (d) or a combination of 10 nM PMA and 10 μg/mL PHA (CD4^+^ T cells) (e). On day 7, 72 h after reactivation, the cells were fixed to visualize vDNA (red, as pointed by red arrow) and vRNA (green) with bDNA imaging. Representative images are shown for the control condition. Nuclei are stained with DAPI (blue), and scale bars represent 10 μm. (f, g) The numbers of vRNA spots per infected cell are shown for the unactivated and reactivated cells, measured with bDNA imaging 7 days posttransduction in the presence of CX014442. The numbers of cells that were imaged and analyzed per condition are shown in Table S2f and S2g. Download FIG S5, TIFF file, 3.2 MB. Copyright © 2022 Janssens et al. 2022 Janssens et al. https://creativecommons.org/licenses/by/4.0/ This content is distributed under the terms of the Creative Commons Attribution 4.0 International license.

10.1128/mbio.00007-22.10 TABLE S4 LEDGIN treatment in primary cells hampers HIV-1 transcription and reactivation from latency. Results of negative binomial regression analysis of the number of vRNA spots per infected cell in PMBCs donor 1 (a) and PBMCs and CD4^+^ T cells donor 2 (b). Parameter estimates are reported, along with the standard error, *P* value, and OR of each parameter. The ORs of vRNA spots per infected cell are shown for unactivated cells (top), the ORs of the interaction term between concentration and reactivation are shown in the middle panel, and the recalculated ORs are shown for the reactivated cells (bottom). OR, odds ratio. Download Table S4, JPG file, 1.2 MB. Copyright © 2022 Janssens et al. 2022 Janssens et al. https://creativecommons.org/licenses/by/4.0/ This content is distributed under the terms of the Creative Commons Attribution 4.0 International license.

The enrichment of vDNA spots was analyzed in more detail by measuring the integrated copies with Alu-LTR qPCR over time. CD4^+^ T cells were transduced with HIV-1 fLuc, and the integrated copies were determined on day 3 and day 7 posttransduction. In the untreated cells and the cells treated with 2.0 μM CX014442, a sharp reduction in integrated copies was observed between day 3 and day 7 posttransduction. However, the number of integrated copies in the cells treated with 4.0 and 8.0 μM CX014442 remained stable over time (Fig. S6a). This suggests a negative selection of integrated copies in the untreated and 2.0 μM CX014442-treated cells and/or a positive selection of integrated copies in the cells treated with higher CX014442 concentrations. To further investigate if the selection of integrated copies might be linked with LEDGIN-induced latency, we determined the integrated copies over time after RAL treatment in parallel (Fig. S6b). Here, all concentrations showed a strong reduction in integrated copies except for the highest concentration of RAL, where integrated copies also remained rather stable over time. In parallel with quantitative PCR (qPCR), the integrated copies were determined with bDNA imaging. Since bDNA imaging does not allow discrimination between integrated and unintegrated vDNA, we only measured the vDNA spots at day 7 posttransduction. bDNA imaging showed clear differences in vDNA spots between CX014442- and RAL-treated cells. CX014442 treatment dose-dependently increased the average vDNA spots per cell measured at day 7 posttransduction, while a reduction was observed in the RAL-treated cells (Fig. S6c), indicating that only CX014442, but not integration inhibitors in general, induces an enrichment of vDNA spots over time in primary cell cultures. These data could point to an enrichment of deeply latently infected cells after transduction of primary cells with a single-round HIV-1 virus and the negative selection of productively infected cells.

10.1128/mbio.00007-22.6 FIG S6 Selection of deep latent HIV-1 provirus in primary cell cultures. (a, b) CD4^+^ T cells were transduced with single-round HIV-1 expressing firefly luciferase (fLuc) in the presence of CX014442 (a) or RAL (b). The integrated copies were determined by Alu-LTR qPCR on day 3 and day 7 posttransduction and normalized to CCR5. (c) Average number of vDNA spots per cell, measured with bDNA imaging 7 days posttransduction in the presence of CX014442 or RAL, shown as mean ± SEM. (d) Absolute number of cells in control or CX014442- or RAL-treated cells. Download FIG S6, TIFF file, 0.3 MB. Copyright © 2022 Janssens et al. 2022 Janssens et al. https://creativecommons.org/licenses/by/4.0/ This content is distributed under the terms of the Creative Commons Attribution 4.0 International license.

## DISCUSSION

Latently infected cells as a source of viral rebound prevent HIV-1 eradication and therefore remain the greatest challenge toward an HIV-1 cure. However, a better understanding of the mechanisms controlling HIV-1 expression and silencing is needed. In recent years, evidence has grown that the site of integration, as well as the chromatin landscape surrounding the integration site, affects the transcriptional state of the provirus. Still, at present, the impact of integration site selection on the establishment and maintenance of the HIV-1 reservoirs remains poorly understood. We have used an *in vitro* latency model to investigate the impact of LEDGIN-mediated retargeting on HIV-1 latency and reactivation with bDNA imaging in comparison to raltegravir (RAL), a clinically approved integrase inhibitor. While *in vitro* latency models cannot fully capture all mechanisms governing HIV-1 latency *in vivo*, they provide us with important insights on how to reduce HIV-1 transcription and reactivation in cell lines and primary cells before this concept moves to a more translational clinical setting. In the present study, the impact of LEDGIN-mediated retargeting on HIV-1 latency and reactivation was investigated with bDNA imaging in comparison to RAL, a clinically approved integrase inhibitor. In contrast to average-based readouts, bDNA imaging is a single-cell technology capable of discriminating between actively (RNA expression) or latently (no vRNA expression) HIV-1-infected cells (Fig. 1c). Using simultaneous detection of integrated vDNA and vRNA expression at the single-cell level, we investigated the role of integration site selection on HIV-1 transcription and reactivation.

### Retargeting HIV-1 integration by LEDGINs reduces the baseline transcriptional state and reactivation of the provirus.

Consistent with previous reports (21, 33), LEDGIN CX014442 and RAL both reduced HIV-1 integration, as evidenced by a dose-dependent reduction in vDNA spots per cell (Fig. 4a and b). Remarkably, only treatment with LEDGINs reduced HIV-1 expression (Fig. 4c, blue spots) and reactivation from latency (Fig. 4c, green spots) in a dose-dependent manner. RAL treatment, on the contrary, only slightly decreased the number of vRNA spots per infected cell in both unactivated and TNF-α-treated cells, indicating that the observed phenotype is not due to reduced integration. Only in unactivated cells treated with a high concentration of RAL (62.3 nM, i.e., 5× IC_50_ value), the odds ratio of vRNA spots per infected cell decreased by 89%. By using a double-reporter virus, an increase in the HIV-1 latent fraction at very high concentrations of RAL was observed before (34) and has been associated with illegitimate integration associated with long terminal repeat (LTR) deletions or duplications or other rearrangements (47). In addition, 16% (16 out of 100 cells) displayed a high-expressor phenotype upon reactivation after treatment with the highest dose of RAL, while this decreased to 3% (3 out of 100 cells) for an equivalent dose of CX014442 (Fig. 4c and d). We also investigated GS-9822, a preclinical LEDGIN candidate with nanomolar potency. GS-9822 likewise reduced HIV-1 transcription and reactivation but at nanomolar concentrations. Collectively, these data strongly indicate that retargeting integration away from preferred integration sites induces a reservoir that is more quiescent and more refractory to reactivation. LEDGIN-mediated retargeting, and not inhibition of HIV-1 integration as such, is responsible for this quiescent phenotype.

LEDGINs silence HIV-1 expression by retargeting the HIV-1 provirus to less transcriptionally active sites in the genome. However, in agreement with earlier data (33), a few high vRNA expressors persist even after a high dose of CX014442 or GS-9822 (Fig. 4c and Fig. 6c), suggesting that LEDGINs alone fail to completely prevent HIV-1 reactivation. We can think of two possible explanations. It is possible that the residual high vRNA expressors after LEDGIN treatment represent rare proviruses that are simply not retargeted by LEDGINs and are still tethered to H3K36me3 by LEDGF/p75. With the barcoded HIV vectors (B-HIVE) technology, it was shown that the proviruses with strongest expression were located closer to H3K36me3 in comparison to all proviruses, supporting the hypothesis that the high expressors are the ones not retargeted by LEDGINs (33). Alternatively, transcription is also stimulated by enhancers (37, 48), and interestingly, LEDGIN CX014442 does not affect the proximity of HIV-1 to these enhancers (33). Irrespective of the LEDGIN concentration, the proviruses with the highest expression level were found closer to the enhancer markers H3K27ac and H3K4me1 (33). If residual transcription after LEDGIN-mediated retargeting is caused by stochastic integration close to (super)enhancers, an additional block in enhancer-stimulated transcription may contribute to a complete suppression of HIV-1 transcription. Therefore, addition of antagonists of BRD4 or MED1, both known for their role in enhancer biology, to LEDGINs may form an optimal latency-promoting agents (LPA) cocktail to completely suppress HIV-1 expression.

### Single-cell imaging to study HIV-1 latency.

In contrast to conventional DNA or RNA fluorescence imaging, branched detection of vDNA and vRNA has an improved signal-to-noise ratio. Hybridization of a minimum of 3 double ZZ probes, covering each a 28-bp nucleotide region, is needed to obtain sufficient signal, reducing nonspecific amplification and ensuring high specificity. On the other hand, sensitivity is increased by an intense signal amplification, ranging between 1,200- and 8,000-fold, depending on the number of double ZZ probes that hybridize to the target sequence. In our hands, labeling of vRNA was highly specific, with no to very low numbers of fluorescent spots detected in nontransduced cells (on average, 0.02 spots per cell in SupT1 cells and no background primary cell lines). However, vDNA labeling was less sensitive and specific. On average, we observed 0.18 spots per cell in nontransduced SupT1 cells and 0.09 spots per cell in nontransduced primary cell lines, which is in line with previous reports (43). Likewise, the sensitivity of the vDNA detection was lower than the vRNA detection since we observed 30% of vRNA^+^ cells in the unactivated control condition in which no vDNA was detected; this percentage increased up to 50% in TNF-α-treated control cells. Incomplete denaturation of double-stranded DNA (dsDNA) prior to hybridization of vDNA to the probes may account for the lower sensitivity, together with the lower accessibility of vDNA in the nucleus. Despite the lower sensitivity of the detection of vDNA than vRNA, we clearly observed a dose-dependent reduction in vDNA upon increasing concentrations of LEDGINs.

Single-cell approaches will become indispensable in the future (49) since bulk approaches are limited in understanding complex biological processes. This is the case for HIV-1 latency, a particularly complex process due to the transcriptional heterogeneity of HIV-1-infected cells (50). In analogy, the B-HIVE technology providing single-provirus resolution allowed correlation of the expression of a single provirus to the integration site and the associated epigenetic features (33). The study provided unambiguous evidence that the integration site directly affects the transcriptional activity of the provirus, which would have been impossible to prove with bulk technologies. Furthermore, the study showed that transcriptionally silent proviruses after LEDGIN treatment are located at an increased distance from epigenetic marks associated with active transcription. Unfortunately, no useful data on reactivation were obtained with barcoded viruses. In the present study and using single-cell imaging, we not only corroborate that the transcriptional state of the provirus depends on the integration site but also demonstrate that retargeted virus is refractory to TNF-α-mediated reactivation. Moreover, single-cell imaging provides information on the three-dimensional location of the provirus; LEDGINs relocate the residual provirus toward the center of the nucleus. Whether this alternative location results from a stochastic, diffusion-controlled event or reflects a redundancy in integration site selection using an alternative host factor (e.g., CPSF6) or is mediated by integrase-dependent targeting (51) remains to be investigated.

### Shock or lock the latent reservoir?

Strategies to eradicate the latent reservoir have mainly been centered on reactivating viral expression of latent HIV-1 in patients. In this so-called shock-and-kill strategy, latency-reversing agents (LRAs) have been investigated for their potential to reactivate proviral transcription in latently infected cells (52, 53). Activation by LRAs is expected to induce viral antigen expression, rendering the HIV-1-infected reservoirs cells susceptible to elimination by cytotoxic T lymphocytes, natural killer cells, or viral-induced cytotoxicity, thereby reducing the size of the latent reservoir. Early treatment initiation studies have shown that reservoir reduction can delay viral rebound (54, 55). However, mathematical models predict that in order to completely prevent viral rebound, the latent reservoir will need to be reduced by 2,000 times or more (56), which remains a significant obstacle considering that several LRA-based clinical trials have shown little to no reduction in reservoir size (53, 57). The limited response of LRAs is partly due to the multiple levels at which HIV-1 gene expression is regulated. The transcriptional state of the provirus is not only regulated by the genomic profile of the host cell but as well by the site of integration and the associated chromatin features (33, 50, 58). This presents a major challenge for the shock-and-kill strategy. Since it is known today that the nearby promoter and enhancer regions influence the reactivation capacity of the provirus (37, 53), the importance of integration sites may have been underestimated in the past. Understanding how the proviral integration site impacts transcription and reactivation is indispensable to design shock-and-kill strategies in which effective LRAs also target the proviruses integrated on the sweet spot between reversible and permanent latency.

Considering the challenges posed by complete eradication of HIV-1, alternative cure strategies are being investigated. Albeit less definite, there is growing interest into a functional cure of HIV-1, which aims at HIV-1 remission or sustained virological control. The block-and-lock strategy is an example of a functional cure strategy that aims to permanently lock the virus into a transcriptionally silent or deep latent state, unable to rebound even in the absence of cART. In the present study, we have shown that LEDGIN-retargeted proviruses display reduced levels of HIV-1 expression and reactivation in T cell lines and in primary cell cultures. As such, retargeting integration out of the preferred sites, thereby forcing the provirus into deep latency, represents a promising block-and-lock approach. Still, as HIV-1 latency is often not permanent but is a reversable process, it is crucial that latency promoting strategies induce a permanent silencing of HIV-1 gene expression, being sustained even after withdrawal of the drug. LEDGIN-mediated retargeting could be used to modify the latent reservoir early during its formation, thereby, at least in theory, inducing an irreversible and long-lasting state of deep latency.

Can we modify the size and rebound dynamics of the latent reservoir with LEDGINs? It is well-known that early treatment can affect the size of the reservoir (54, 55). In addition, early results from the eCLEAR trial show that the size and rebound dynamics of the latent HIV-1 reservoir can be pharmacologically modified at the moment of treatment initiation in treatment-naive patients (59). Addition of LEDGINs during early treatment may affect the functional reservoir, and LEDGINs would also be beneficial as part of preexposure prophylaxis to ensure that any residual integration will result in provirus in a deep sleep. With regard to chronically infected patients, recent studies suggest that the majority of the reservoir responsible for HIV-1 rebound might only be established during cART initiation (60, 61), implying that LEDGINs could also have a role in the first-line treatment of patients diagnosed years after infection. Lastly, whether LEDGINs can still modify the latent reservoir in chronically infected patients after long-term ART remains unknown. This should first be investigated in relevant animal models by treatment interruptions to reactivate and retarget the replication-competent HIV-1 reservoir. If positive results are obtained, well-designed clinical trials will be necessary to evaluate if treatment interruptions can mobilize and modify the already formed reservoir in chronically infected patients. LEDGINs are now advancing into clinical trials as allosteric integrase inhibitors, which will facilitate their translation into clinical trials for an HIV-1 cure (62).

The debate is open on whether we should shock or lock the latent reservoir. Although both approaches differ in their objectives, they are both dependent on integration site distribution. While the shock-and-kill approach is limited by integration into nonpermissive sites, this feature is exploited by the LEGDIN-mediated block-and-lock strategy that aims to redirect the integration toward those sites in order to obtain the permanently silenced reservoir. Finally, the combination of “lock and shock” presents an alternative, yet unexplored, strategy. Addition of LEDGINs to early treatment protocols may already reduce the size of the functional reservoir. Afterward, residual replication-competent virus prone to rebound upon treatment interruption (e.g., integrated close to enhancers) could be eradicated with potent LRAs. Either way, an increased understanding of the impact of integration site selection on HIV-1 transcription and reactivation will be important for future cure approaches.

### Does retargeting select deep latent provirus?

Our study also points to the accumulation of LEDGIN-retargeted, but transcriptionally silent, provirus in primary cell culture. Consistent with this observation, it has been shown that the lack of HIV-1 gene expression, either in uninfected or latently infected cells, is associated with a proliferative advantage and cell survival (58). Therefore, the decrease in integrated copies in cells transduced with a single-round HIV-1 virus over time is most likely due to outgrowth of the uninfected cell population. It implies that transduced cells are negatively selected for. Although the HIV-1 virus used was Nef deleted, other viral accessory proteins are known to induce cellular toxicity (63, 64). By retargeting and inducing transcriptionally silent provirus, transduced cells may lose their selection disadvantage and grow at the same rate. As a result, the level of integrated vDNA will increase dose dependently with the LEDGIN concentration. In agreement, no large reductions in absolute cell number were observed, excluding toxicity due to HIV-1 gene expression in the cells not treated with LEDGINs (see Fig. S6d in the supplemental material). Selective survival of infected cells with retargeted provirus after LEDGIN treatment caused by the lack of HIV-1 gene expression seems paradoxical with the goal of obtaining an HIV-1 cure. However, recent studies on integration sites of proviruses in elite controllers (EC), individuals who spontaneously control HIV-1 without treatment, are consistent with our findings *in vitro* (38). In ECs, intact provirus tends to be inserted at noncoding regions, such as centromeric DNA and dense heterochromatin gene deserts, regions less permissive for active viral transcription. The data suggest that cell-mediated immune responses induce a positive selection of proviral sequences with features of deep latency (38, 41). Similar observations were recently made in a follow-up study by Einkauf et al. using longitudinal evaluations of integrated proviruses (65). That study shows an accumulation of transcriptionally silent proviruses over time integrated into heterochromatin regions (i.e., nongenic and satellite DNA) in patients on long-term cART. These clinical data exemplify that persistence of deeply latent provirus is no major concern for block-and-lock functional cure strategies. As such, ECs and patients on long-term cART provide translational evidence for a LEDGIN-induced block-and-lock functional HIV-1 cure. Can LEDGINs accelerate the natural block-and-lock phenotype observed in patients? When a safe and potent LEDGIN as an allosteric integration inhibitor is approved, this question can be addressed by adding LEDGINs to cART preferentially during acute infection when the reservoir is still formed.

In conclusion, a single-cell-based bDNA imaging approach was established to study the link between integration and transcription at the single-cell level. This study demonstrates that retargeting integration affects the transcription and reactivation of HIV-1. In terms of an HIV-1 cure, the link between integration and transcription will be crucial to address in any cure strategy. LEDGIN-mediated retargeting into transcriptionally silent sites severely hampered HIV-1 transcription and reactivation and provides the rationale for a block-and-lock functional cure strategy based on retargeting the proviral integration. This bDNA imaging technology can be further extended to evaluate other block-and-lock cure approaches aimed at HIV-1 remission.

## MATERIALS AND METHODS

### Cell culture.

All cells were tested to be mycoplasma free (PlasmoTest; InvivoGen) and cultured in a humidified atmosphere of 5% CO_2_ and 37°C. SupT1 cells were provided by the National Institutes of Health (NIH) AIDS reagent program. They were cultured in RPMI 1640 (Gibco) supplemented with 10% (vol/vol) fetal bovine serum (FBS; Gibco) and 0.01% (vol/vol) gentamicin (Gibco).

### Compounds.

All compounds were diluted in dimethyl sulfoxide (DMSO) and stored at room temperature (RAL) or at −20°C (LEDGIN) to ensure compound stability. The IC_50_ value was calculated for each compound based on the reduction in firefly luciferase reporter expression in a single-round infection assay (see Fig. S1 in the supplemental material). The LEDGIN CX014442 has been characterized extensively in the past (24), and its IC_50_ was determined here at 9.6 μM using the fLuc activity as readout. Hence, concentrations of 3.12, 6.25, 12.5, 25.0, and 50.0 μM CX014442 correspond to 0.32×, 0.65×, 1.3×, 2.6×, and 5.2× the IC_50_, respectively. The 50% cytotoxic concentration (CC_50_) of CX014442 for MT-4 cells has been previously determined at 96.0 μM ± 16.0 (24). For the experiments shown in Fig. S6, CX014442 was freshly diluted in DMSO, and the IC_50_ was determined at 3.1 μM. Hence, concentrations of 2.0, 4.0, and 8.0 μM correspond to 0.65×, 1.3×, and 2.6× the IC_50_, respectively. The control integrase strand transfer inhibitor raltegravir (RAL) was provided by the NIH AIDS Research and Reagent Reference program, and its IC_50_ was determined at 12.9 nM. Therefore, the concentrations of 3.9, 7.8, 15.6, 31.2, and 62.3 nM RAL were used that correspond to 0.32×, 0.65×, 1.3×, 2.6×, and 5.2× the IC_50_, respectively. The LEDGIN GS-9822 has been characterized previously (34), and its IC_50_ was determined at 17.6 nM. Therefore, concentrations of 22.8, 45.7, and 91.4 nM GS-9822 were used that correspond to 1.3×, 2.6×, and 5.2× the IC_50_, respectively.

### HIV-1 fLuc activity assay.

The viral molecular clone pNL4-3.Luc.RE- was obtained through the NIH AIDS Research and Reagent Reference program and was used to produce HIV-1 replication-deficient luciferase reporter virus, further referred to as HIV-1 fLuc. Replication-deficient (also referred to as “single-round”) virus was prepared by cotransfection of HEK-293T cells with pNL4-3.Luc.RE- and pVSV-G. The cells were transduced with HIV-1 fLuc and harvested at distinct days posttransduction. The cells were washed, lysed in buffer (50 mM Tris, 200 mM NaCl, 0.2% [vol/vol] NP-40, and 5% [vol/vol] glycerol) and subsequently analyzed for firefly luciferase activity (One-Glo; Promega GMBH, Mannheim, Germany). Chemiluminescence was measured with a Glomax luminometer (Promega). Readouts were normalized for protein content as determined by the bicinchoninic acid (BCA) assay (Thermo Scientific Pierce).

### Transduction and reactivation of SupT1 cells.

We transduced 2.0 × 10^5^ SupT1 cells in a 48-well plate with 1.4 × 10^4^ pg p24 HIV-1-fLuc, and they were grown in the presence of different inhibitors described above. On day 3, a sample was collected for the luciferase reporter assay (fLuc activity), and the cells were washed extensively to remove cell-free virus and compounds. The cells were kept in culture until reseeding in a 12-well plate on day 6, followed by further culturing until day 10 to allow silencing of the HIV-1 gene expression. On day 10, the cells were either left untreated as control or were reactivated with 10 ng/mL tumor necrosis factor alpha (TNF-alpha; Immunosource, Zoersel, Belgium). Samples for bDNA imaging and the fLuc activity assay were taken on day 11, 24 h after reactivation. The timeline of the transduction and reactivation experiments is outlined in Fig. 1d.

### Isolation of primary cells.

Human peripheral blood mononuclear cells (PBMCs) were isolated from buffy coats obtained from the Red Cross Blood Transfusion Center (Mechelen, Belgium) by using a Lymphoprep density gradient (Stem Cell Technologies, Cologne, Germany) and further stored in liquid nitrogen until use. CD4^+^ T cells were enriched from PBMCs by the bispecific monoclonal antibody CD3/8 provided by the NIH AIDS Research Reference program (BsMAb; 0.5 μg/mL, anti-CD3-anti-CD8) and purified using a custom-made Easysep negative selection kit (Stem Cell Technologies, #19052). The experiments with human blood cells received bioethical approval by the Medical Ethics committee of the KU Leuven (S58969-IRB00002047).

### Infection and activation of primary cells.

PBMCs were thawed from liquid nitrogen and activated for at least 48 h with 100 U/mL interleukin 2 (IL-2; Peprotech, London, UK) and 10 μg/mL phytohemagglutinin (PHA; Sigma) prior to transduction. The cells were transduced with 1.7 × 10^6^ pg HIV-1 fLuc and cultured in RPMI medium with 15% (vol/vol) FBS and 0.1% gentamicin, supplemented with 10 U/mL IL-2 (PBMCs) or 1 U/mL IL-2 (CD4^+^ T cells) in the presence of different compounds as described above. After collecting a sample for the fLuc activity assay on day 4, the cells were washed twice and either left untreated or activated with 100 nM phorbol myristate acetate (PMA; Sigma) or a combination of 10 nM PMA and 10 μg/mL PHA for PBMCs and CD4^+^ T cells, respectively. The cells were fixed for bDNA imaging 72 h after activation.

### bDNA imaging.

bDNA imaging of viral DNA (vDNA) and viral RNA (vRNA) was performed with RNAscope technology (66) (Advanced Cell Diagnostics) according to the protocol optimized by Puray-Chavez et al. (43). After reactivation, SupT1 cells were allowed to adhere to a poly-d-lysine coverslip (Neuvitro), while primary cells adhered to a polyethyleneimine (PEI)-laminin-coated coverslip (Neuvitro) for 20 min. Afterward, the cells were fixed with 4% (vol/vol) paraformaldehyde and dehydrated using increasing concentrations of ethanol (0, 50, 70, and 100% [vol/vol]). After dehydration, samples were stored in 100% ethanol at −20°C. The cells were rehydrated with decreasing concentrations of ethanol (70, 50, and 0% [vol/vol]) prior to permeabilization with 0.1% Tween 20 (vol/vol). vDNA was detected with a sense probe hybridizing to the antisense region of the HIV-1 genome (*Gag*/*Pol* probe; catalog no. 317701-C1; Advanced Cell Diagnostics). vRNA was detected with an antisense probe targeting the sense non-*Gag*/*Pol* region of HIV-1 (non-*Gag*/*Pol* probe; catalog no. 317711-C2; Advanced Cell Diagnostics). For fluorescence imaging, the probes were conjugated to the Atto 550 fluorophore (vDNA; pseudocolored red in the images) or the Atto 635 fluorophore (vRNA; pseudocolored green in the images), and DAPI (0.001 μg/μL) was used for nuclear staining (blue). The cells were imaged using a laser scanning microscope (Fluoview FV1000; Olympus, Tokyo, Japan), and the images were acquired with the UPLSAPO 60× water-immersion objective (SupT1 cells) or the UPLSAPO 100× oil immersion objective (primary cells) using the DM405/488/559/635 polychromic excitation mirror (Olympus). Three-dimensional stacks were acquired with 0.3-μm step size and 4-μs/pixel sampling speed.

### Alu-LTR qPCR.

Integrated HIV-1 DNA was quantified using a nested real-time Alu-LTR qPCR, performed as described previously (32). We lysed 5 × 10^5^ transduced SupT1 cells or PBMCs in 50 μL of lysis buffer for 1 h at 56°C (10 mM Tris-HCl, pH 8, 1 mM EDTA, 0.01% Triton X-100, and 0.8 mg/mL proteinase K [PK]). The first-round PCR mixture consisted of 5 μL of DNA from lysed cells, 10 μL of iQ supermix (Bio-Rad, Temse, Belgium), 1 μL of each primer (5 μM; Alu forward, gctaactagggaacccactgctta; Alu reverse, tgctgggattacaggcgtgag; and HIV-1 LTR forward, tcccagctactggggaggctgagg), and 2 μL of water. Cycling conditions for the first-round PCR were 95°C for 10 min, followed by 15 cycles of 95°C for 30 s, 60°C for 40 s, and 72°C for 3.5 min. All samples were run at least in duplicate. Five microliters of the first-round product were added to a second-round PCR mixture containing 10 μL of iQ supermix, 1 μL of forward and reverse primer (5 μM; HIV-1 LTR forward, agcttgccttgagtgcttcaa; HIV-1 LTR reverse, tgactaaaagggtctgagggatct), 1 μL of the TaqMan probe (5 μM; ttaccagagtcacacaacagacgggca), and 2 μL of water. The second-round qPCR was performed in a CFX96 (Bio-Rad) for 5 min at 95°C, followed by 45 cycles of 95°C for 15 s, 60°C for 30 s, and 72°C for 1 min. Integrated copies were normalized for input DNA by a parallel CCR5 qPCR as previously described (32). Data were analyzed using the provided CFX96 Bio-Rad software.

### Viral DNA and viral RNA particle detection.

An in-house MATLAB routine (MathWorks) was used for vDNA and vRNA particle detection. For the detection of vDNA and vRNA particles, a Gaussian filter (band-pass filter) was first applied to the raw images for an improved contrast image for further analysis, which is a common procedure for detection of particles that are imaged using a diffraction-limited microscope (optical resolution of 200 to 300 nm). This smoothening band-pass filter improves the signal-to-noise ratio of the image for identification of single particles. Next, a home-written MATLAB routine adapted from Crocker et al. (67) was used for particle detection in the filtered images. Particles were detected per measured z-stack based on an intensity threshold and particle diameter in pixels. Particles that were detected in multiple consecutive z-stacks were considered unique particles if detected in the same *xy* position. The boundary of imaged cells in a single frame was manually drawn.

### Statistical analysis.

Data were analyzed using GraphPad Prism software version 9.00 for macOS (GraphPad). The IC_50_ value of each compound was calculated via a nonlinear regression curve fit of a dose-response curve. A Kruskal-Wallis test was used to test for statistically significant differences between the vDNA spots per cell. SPSS software was used to perform the negative binomial probability distribution. The deviance (goodness-of-fit coefficient) was determined at 1.058 (CX014442, SupT1 cells), 1.474 (RAL, SupT1 cells), 1.168 (GS-9822, SupT1 cells), 1.085 (CX014442, PBMCs donor 1), 1.075 (CX014442, PBMCs donor 2), and 1.118 (CX014442, CD4^+^ T cells donor 2).
