# Supplementary figures and images for: Single-Cell Imaging Shows That the Transcriptional State of the HIV-1 Provirus and Its Reactivation Potential Depend on the Integration Site
Source: mBio. 2022 Jun 16;13(4):e00007-22. doi: 10.1128/mbio.00007-22 (PMC9426465; doi:10.1128/mbio.00007-22)

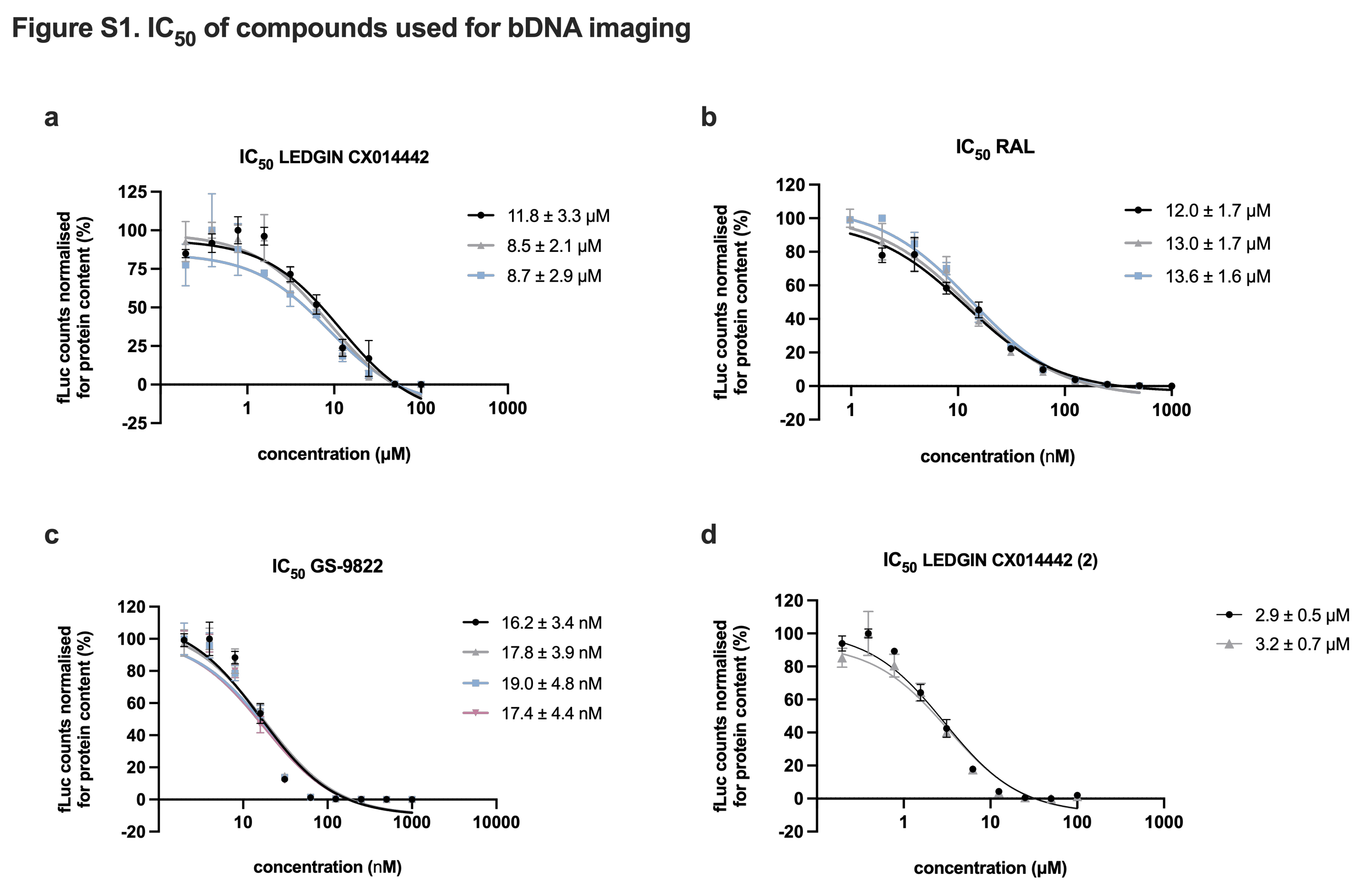

Supplement: FIG S1 [file mbio.00007-22-s0001.tif]

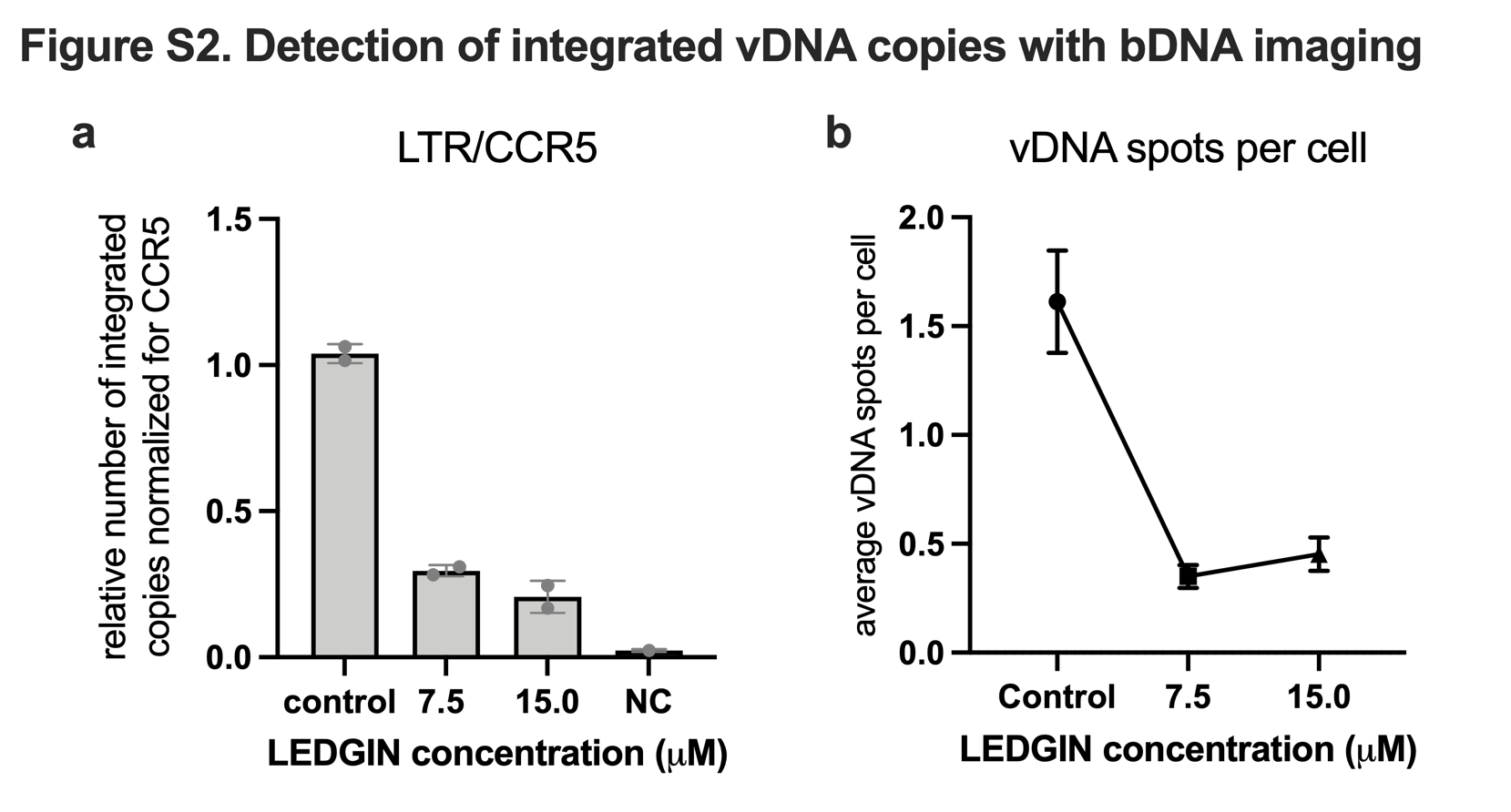

Supplement: FIG S2 [file mbio.00007-22-s0002.tiff]

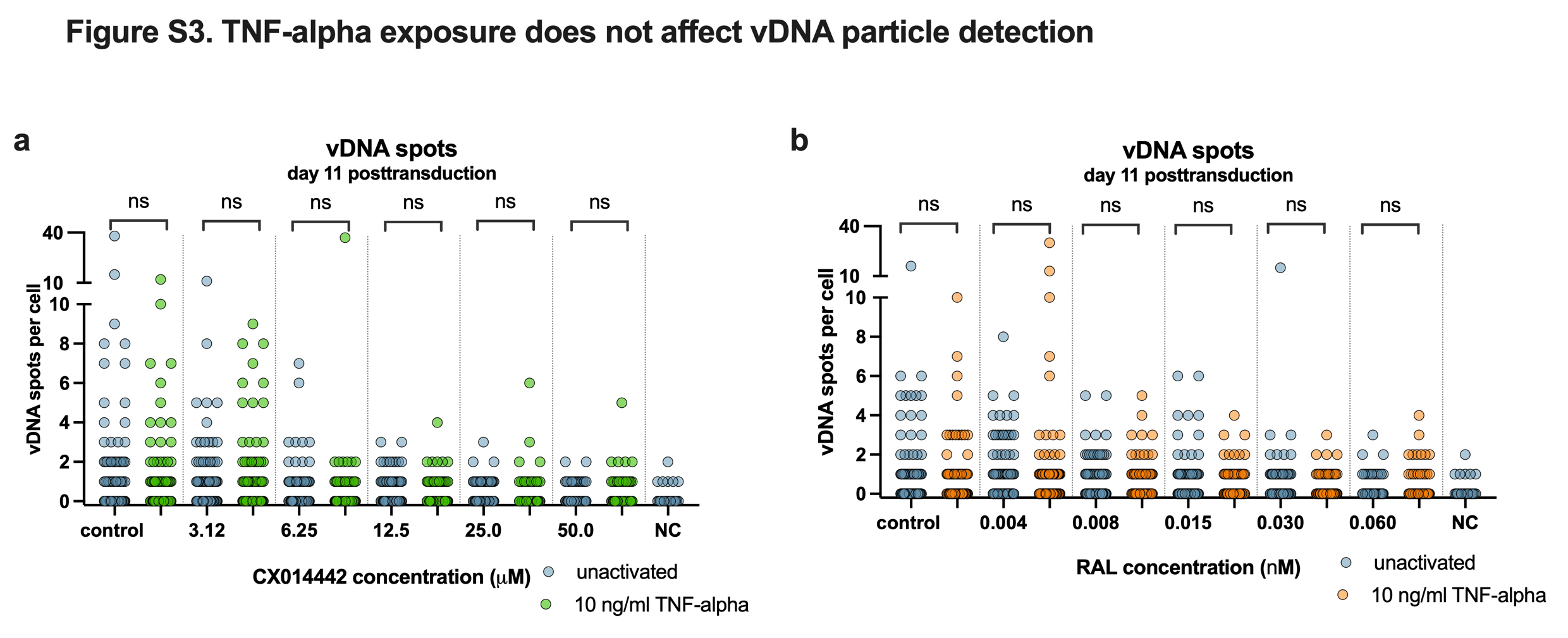

Supplement: FIG S3 [file mbio.00007-22-s0003.tiff]

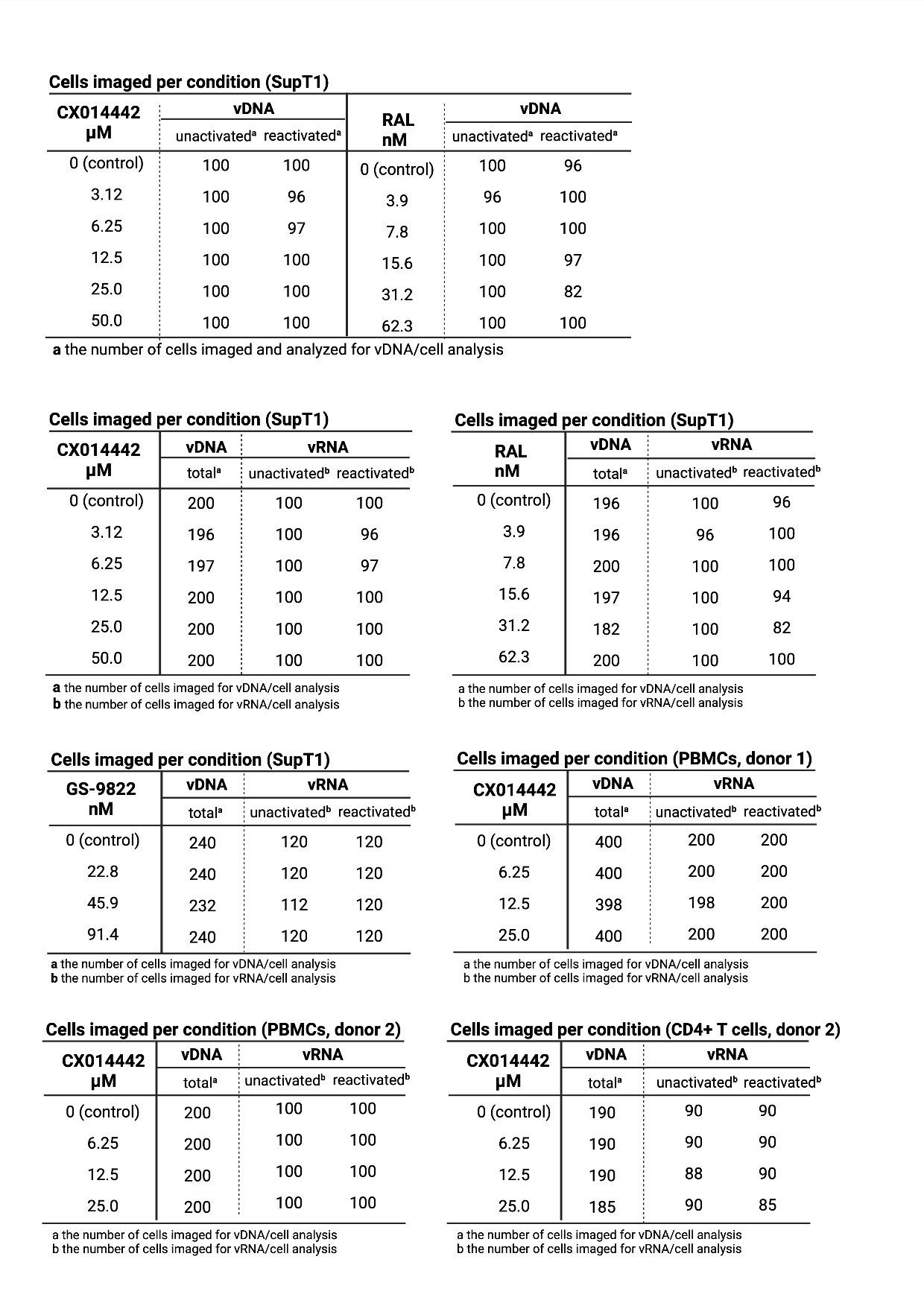

Supplement: TABLE S2 [file mbio.00007-22-s0008.jpg]

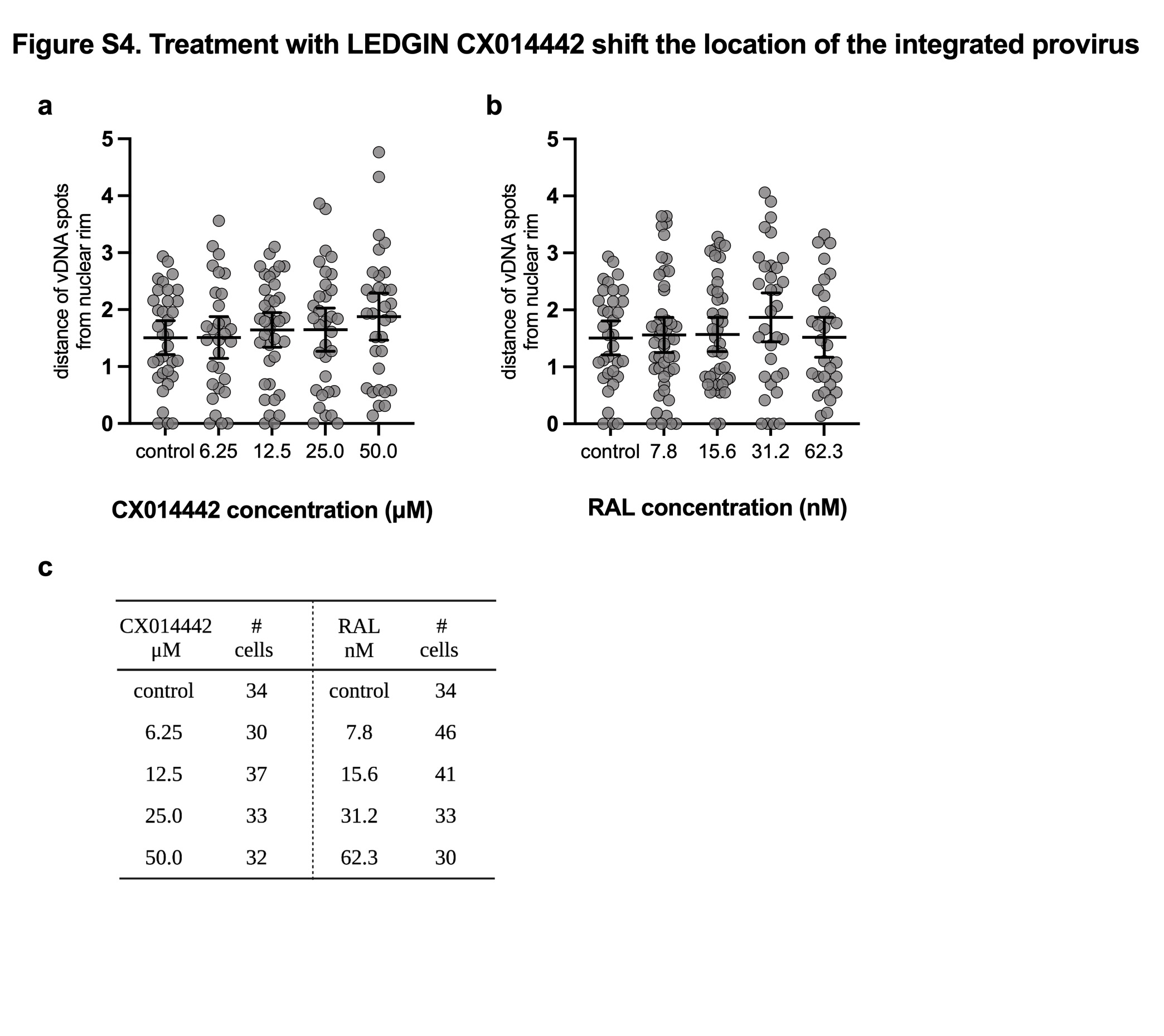

Supplement: FIG S4 [file mbio.00007-22-s0004.tif]

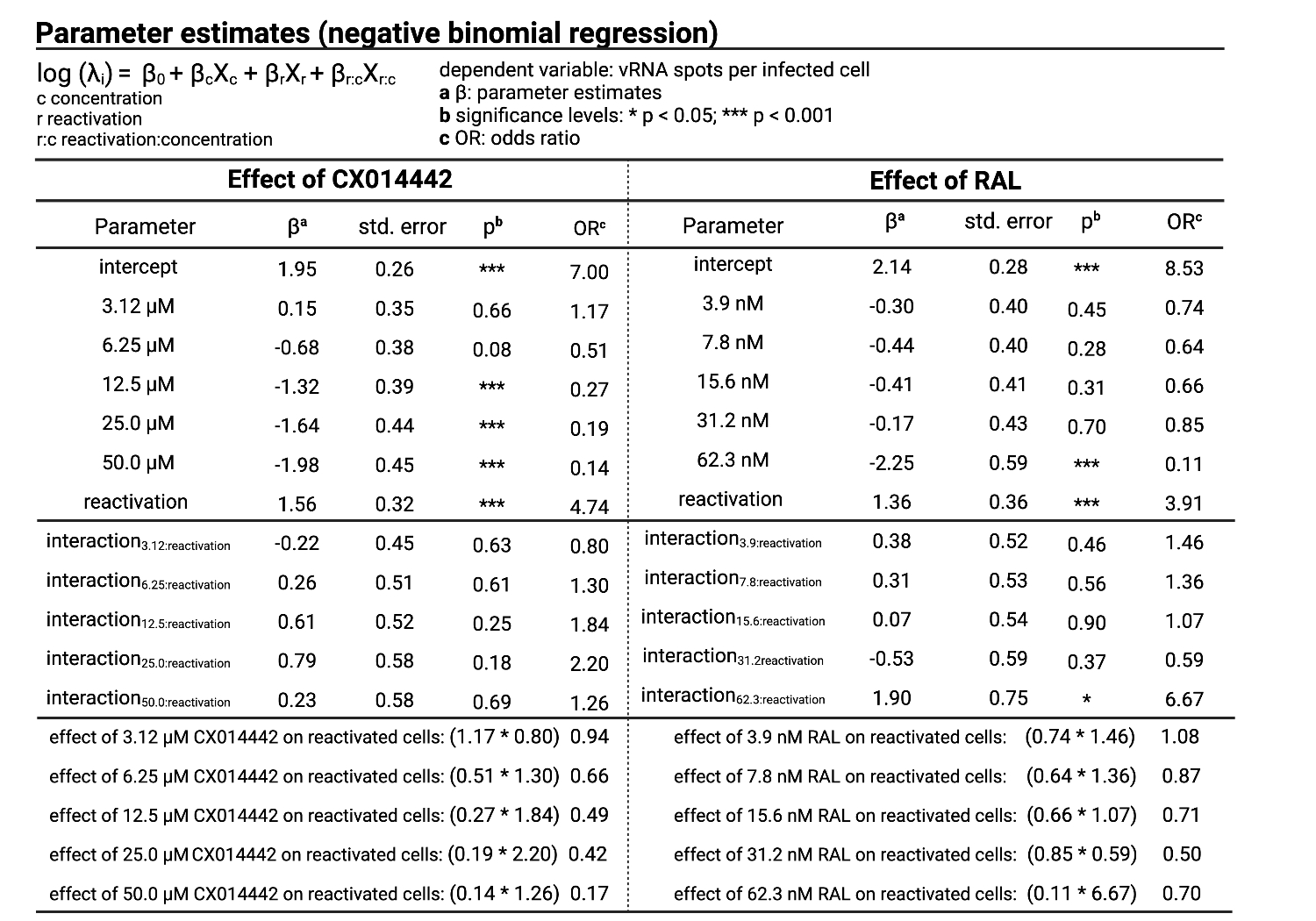

Supplement: TABLE S1 [file mbio.00007-22-s0007.jpg]

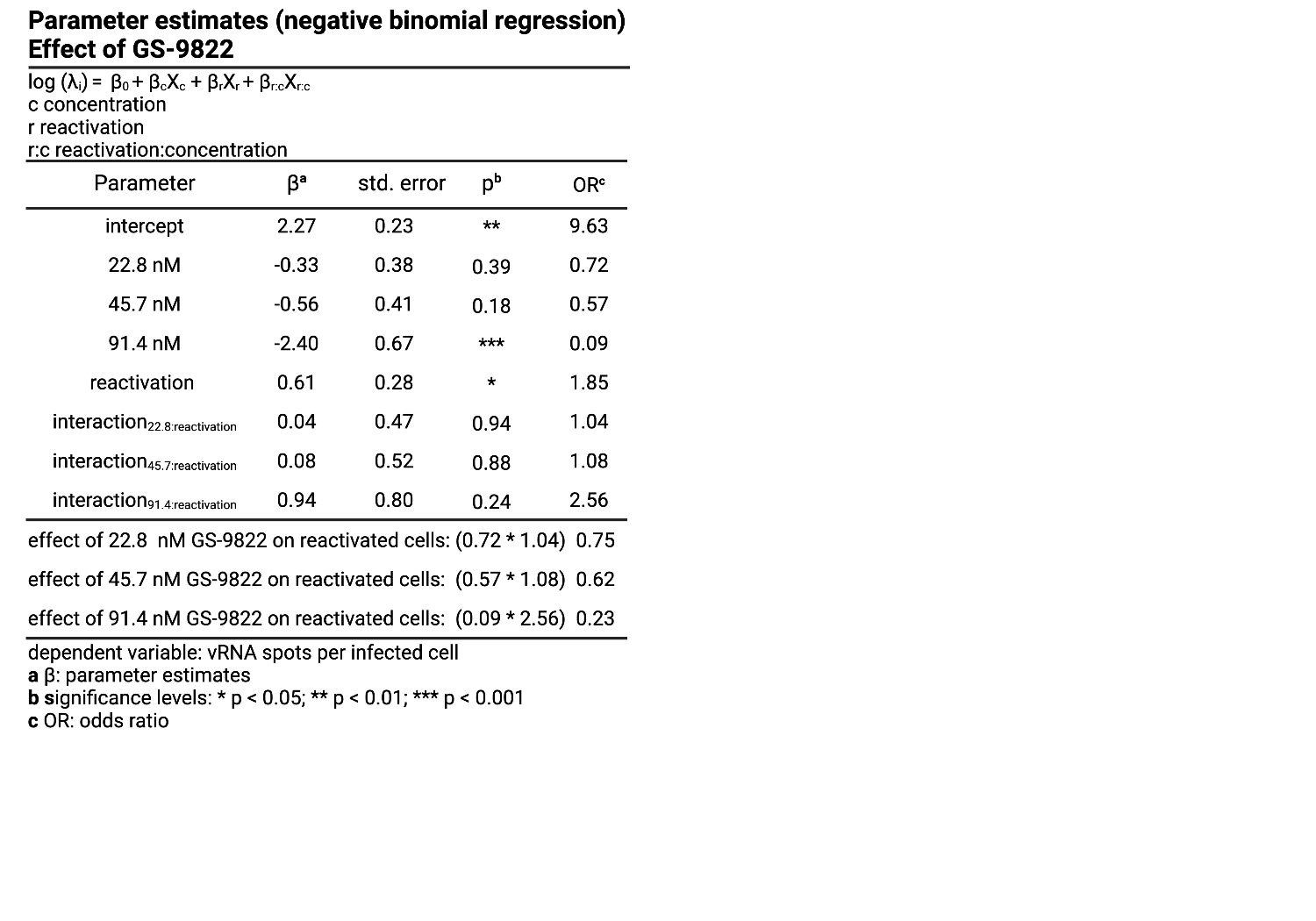

Supplement: TABLE S3 [file mbio.00007-22-s0009.jpg]

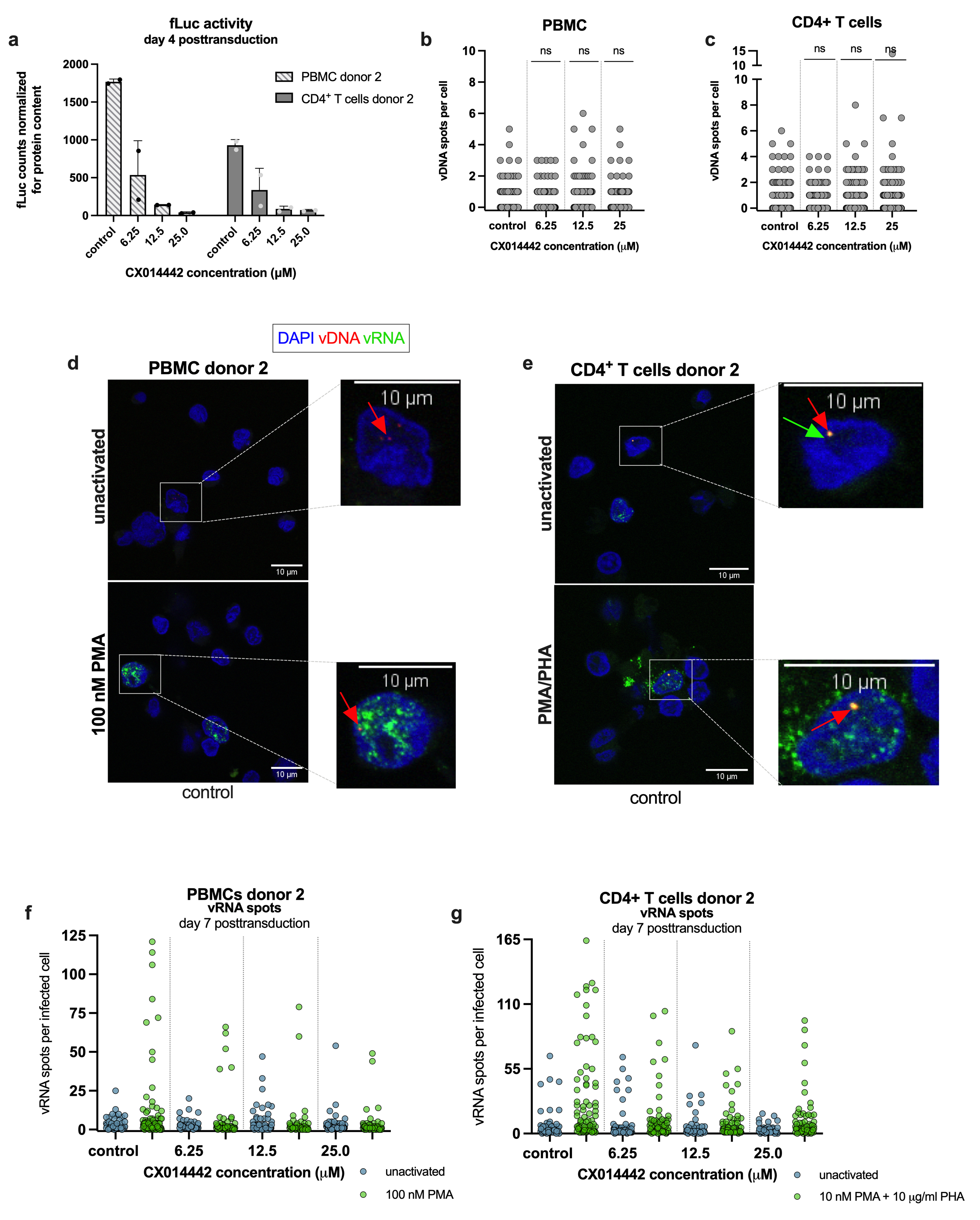

Supplement: FIG S5 [file mbio.00007-22-s0005.tiff]

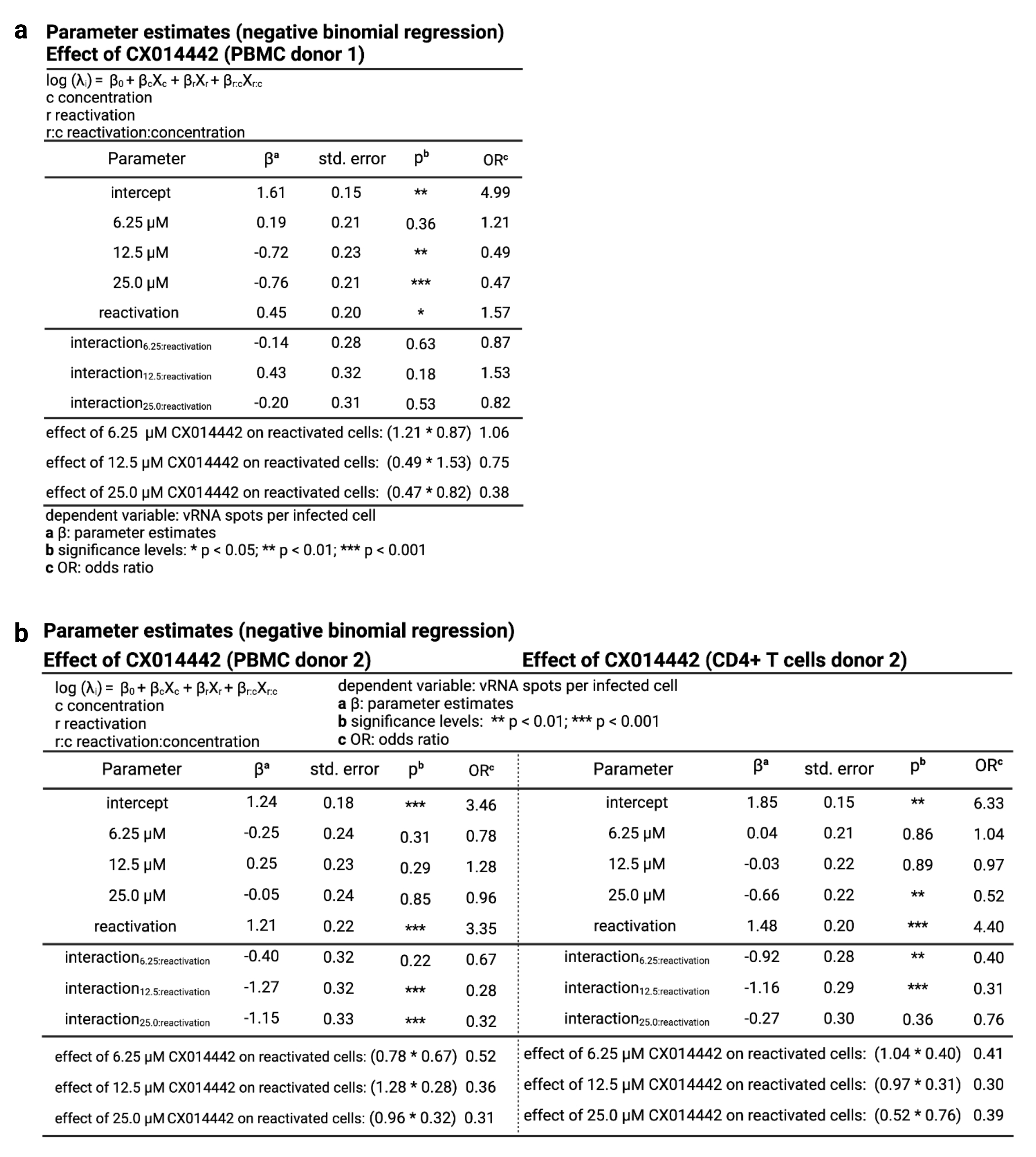

Supplement: TABLE S4 [file mbio.00007-22-s0010.jpg]

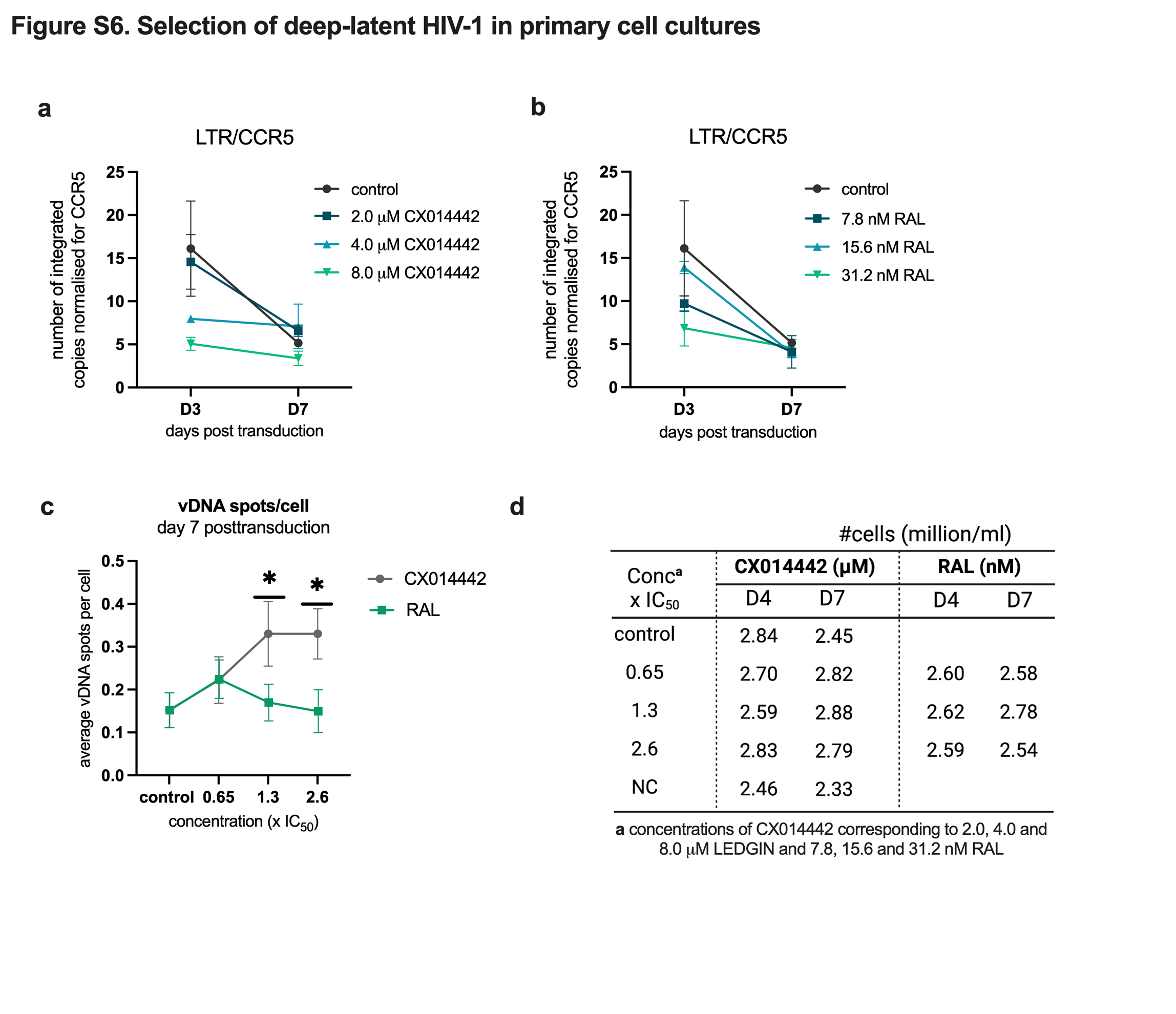

Supplement: FIG S6 [file mbio.00007-22-s0006.tiff]
